# Chemical hypoxia induces apoptosis of human pluripotent stem cells by a NOXA-mediated HIF-1α and HIF-2α independent mechanism

**DOI:** 10.1038/s41598-020-77792-7

**Published:** 2020-11-26

**Authors:** Luciana Isaja, Sofía Mucci, Jonathan Vera, María Soledad Rodríguez-Varela, Mariela Marazita, Olivia Morris-Hanon, Guillermo Agustín Videla-Richardson, Gustavo Emilio Sevlever, María Elida Scassa, Leonardo Romorini

**Affiliations:** Laboratorios de Investigación Aplicada en Neurociencias (LIAN-CONICET), Fundación para la Lucha contra las Enfermedades Neurológicas de la Infancia (Fleni), Ruta 9, Km 52.5, B1625XAF Belén de Escobar, Provincia de Buenos Aires Argentina

**Keywords:** Biochemistry, Cell biology, Developmental biology, Stem cells

## Abstract

Human embryonic and induced pluripotent stem cells (hESCs and hiPSCs) are self-renewing human pluripotent stem cells (hPSCs) that can differentiate to a wide range of specialized cells. Notably, hPSCs enhance their undifferentiated state and self-renewal properties in hypoxia (5% O_2_). Although thoroughly analyzed, hypoxia implication in hPSCs death is not fully determined. In order to evaluate the effect of chemically mimicked hypoxia on hPSCs cell survival, we analyzed changes in cell viability and several aspects of apoptosis triggered by CoCl_2_ and dimethyloxalylglycine (DMOG). Mitochondrial function assays revealed a decrease in cell viability at 24 h post-treatments. Moreover, we detected chromatin condensation, DNA fragmentation and CASPASE-9 and 3 cleavages. In this context, we observed that *P53*, *BNIP-3*, and *NOXA* protein expression levels were significantly up-regulated at different time points upon chemical hypoxia induction. However, only siRNA-mediated downregulation of NOXA but not HIF-1α, HIF-2α, BNIP-3, and P53 did significantly affect the extent of cell death triggered by CoCl_2_ and DMOG in hPSCs. In conclusion, chemically mimicked hypoxia induces hPSCs cell death by a NOXA-mediated HIF-1α and HIF-2α independent mechanism.

## Introduction

Human embryonic stem cells (hESCs) and human induced pluripotent stem cells (hiPSCs) are self-renewing pluripotent stem cells (hPSCs) that can differentiate and give rise to all cell types derived from the three germ layers, endoderm, mesoderm and ectoderm. Particularly, hESCs are derived from the inner cell mass of the human blastocyst, at the early stages of embryonic development^1^. Instead, hiPSCs are reprogrammed from adult somatic cells for example by in vitro transfection of vectors encoding crucial pluripotency transcription factors (e.g. *OCT-4*, *SOX-2*, *KLF-4* and *c-MYC*)^2^. Therefore, hPSCs hold great promise as models for the study of human development and disease, as well as for drug discovery and cell-replacement therapies^1,3^. However, although hPSCs can indefinitely proliferate in vitro they are endowed with a cell-intrinsic property termed mitochondrial priming that renders them highly sensitive to apoptotic stimuli^4^. Thus, all attempts to define the mechanisms that govern cell survival in hPSCs and their differentiating progeny could help to improve large-scale in vitro expansion of hPSCs, the generation of a safe transplantable cell source with minimal or no risk for tumor/teratoma formation, and the clinical outcome of therapies relying on hPSCs-derived cells.

Hypoxia is a pathophysiological condition which is accompanied by an increase in reactive oxygen species (ROS) thus provoking oxidative stress^5^. However, although hypoxia is thought to be a pathological phenomenon, embryonic tissues develop in a hypoxic environment at the early stages, until the cardiovascular and hematopoietic systems are sufficiently differentiated to provide oxygen for them^6^. Upon exposure to hypoxic conditions, cells mount a physiological response to ensure sufficient levels for oxygen-dependent processes. This adaptive response is mainly mediated through the activation of the oxygen-sensitive transcription factors, hypoxia-inducible factors (HIF-1α, HIF-2α/EPAS1 and HIF3-α). In normoxia, HIF-1α and HIF-2α undergo prolyl hydroxylation, which leads to specific binding to the ubiquitin E3 ligase Von Hippel-Lindau, polyubiquitination, and proteasomal degradation. However, HIF-1α and HIF-2α are stabilized in low oxygen levels, dimerize with HIF-1β, which is also known as aryl-hydrocarbon-receptor nuclear translocator, and fine-tune the transcription of target genes^7,8^.

Interestingly, in vitro exposure of hPSCs to reduced levels of oxygen (5%) favors the maintenance of the undifferentiated state, as well as promotes self-renewal and prevents spontaneous differentiation^9–11^. Despite this, hPSCs in vitro culture is still usually performed under ambient air (O_2_ concentration of approximately 21%) enriched with 5% CO_2_, given that modular hypoxia incubator chambers are not frequently available in many cell culture facilities. To overcome this limitation, chemical compounds that inhibit prolyl hydroxylase domain-containing enzymes, like cobalt chloride (CoCl_2_) and dimethyloxalylglycine (DMOG) can be used in vitro to mimic hypoxic conditions by stabilizing HIF-1α and generating ROS^12–14^. In this regard, until the present, the effects of chemical induced hypoxia on hPSCs viability have not been studied. It has been reported that in mouse embryonic stem cells (mESCs) CoCl_2_ induces apoptosis and necrosis through the mitochondria- and death receptor-mediated pathways^12^.

In the present work, we found that chemically mimicked acute hypoxic conditions, induced with CoCl_2_ and DMOG treatments, decreased hPSCs viability. Importantly, at 24 h post-chemical hypoxia induction we observed the appearance of apoptotic hallmarks such as cell ballooning and detachment, pyknotic hyperchromatic nuclei, prominent internucleosomal DNA fragmentation and caspases activation. Remarkably, cell death occurred independently of HIF-1α, HIF-2α and P53 expression levels. However, specific siRNA-mediated downregulation of the pro-apoptotic factor NOXA protected hPSCs from chemical hypoxia induced apoptosis. Collectively, herein we demonstrated that chemical hypoxia triggered apoptosis in hPSCs via a NOXA-mediated HIF-1α and HIF-2α independent mechanism.

## Results

### ***Chemical hypoxia induced by CoCl***_***2***_*** and DMOG stabilizes HIF-1***α*** in hPSCs***

Hypoxia was induced in H9 hESCs and FN2.1 hiPSCs grown on Vitronectin coated cell culture dishes with fully defined Essential E8 medium (E8) by a chemical method. Particularly, we mimicked hypoxic conditions in hPSCs using the following chemicals: CoCl_2_ (250 µM for 24 h) and DMOG (1 mM for 24 h). Both treatments caused an increase in the intracellular levels of HIF-1α, which was analyzed by Western blot (Supplementary Fig. S1a). In the case of HIF-2α we determined basal expression levels that were not induced upon chemical hypoxia stimulus (Supplementary Fig. S1a). Moreover, hypoxic conditions were further validated by analyzing the mRNA expression levels of BCL2/adenovirus E1B 19 kDa interacting protein 3 (*BNIP-3*), a well-known transcriptional target of the HIF-1α/HIF-1β complex^15^ (Supplementary Fig. S1b). Interestingly, concentrations of CoCl_2_ as low as 50 µM were able to increase significantly *BNIP-3* mRNA expression levels in H9 hESCs (Supplementary Fig. S1c).

### Chemical hypoxia triggers apoptosis of hPSCs

**Figure 1:**
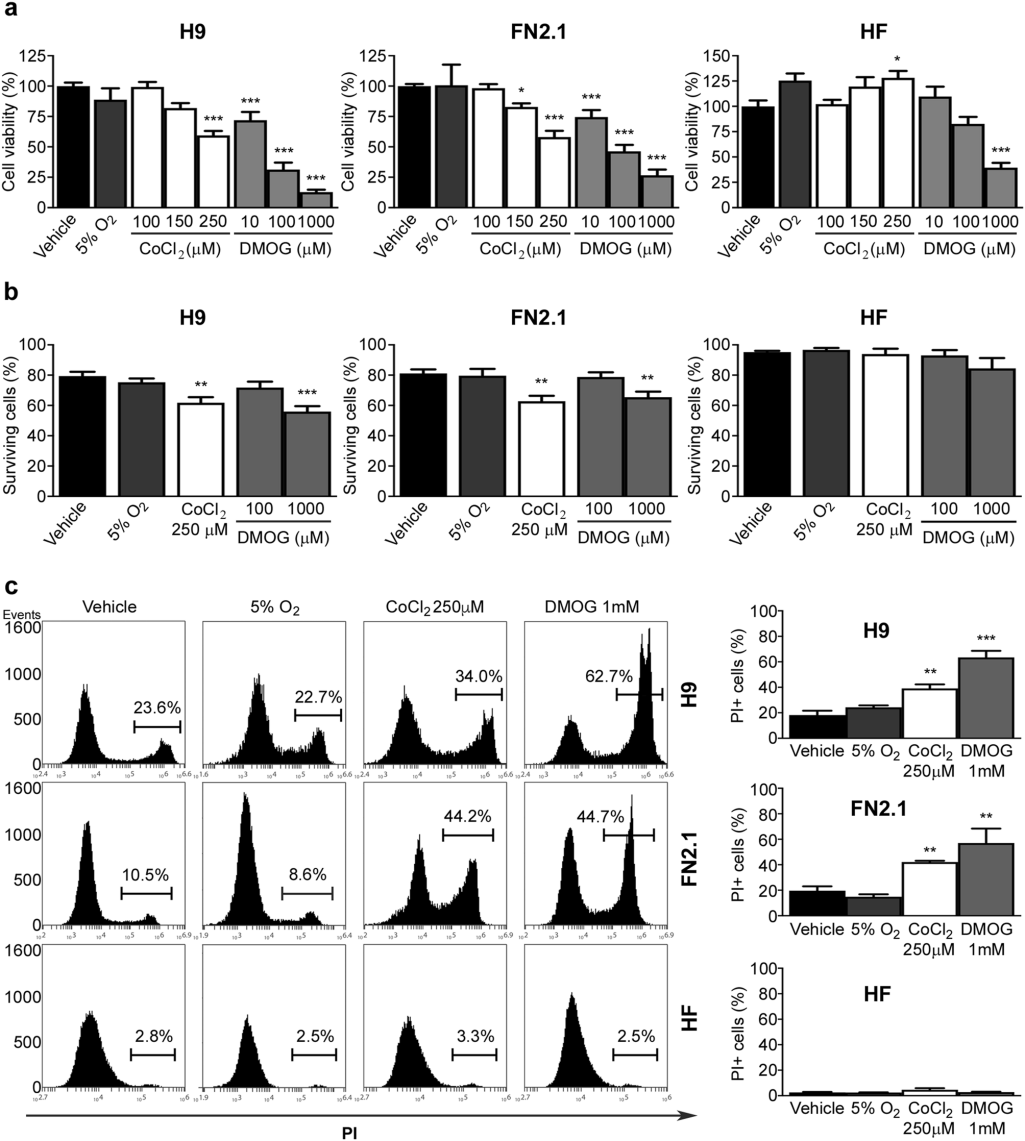
Changes in cell viability and cell death induced by hypoxic conditions in hPSCs and HF. (**a**) H9, FN2.1 and HF cell viability was analyzed 24 h post-treatment with 5% O_2_ or increasing concentrations of CoCl_2_ and DMOG by XTT colorimetric assay. Vehicle = DMSO. Mean + SEM from three independent experiments are shown. Statistical analysis was done by one-way ANOVAs followed by Dunnett's multiple comparisons test, (*) *p* < 0.05 and (***) *p* < 0.001 vs. Vehicle. (**b**) Bar graphs show percentage of surviving cells assessed by Trypan blue exclusion method 24 h after incubation with 5% O_2_, CoCl_2_ (250 µM) and DMOG (100 µM and 1 mM). Mean + SEM from at least three independent experiments are shown. Statistical analysis was done by one-way ANOVAs followed by Dunnett's multiple comparisons test, (**) *p* < 0.01 and (***) *p* < 0.001 vs. Vehicle (DMSO). (**c**) Representative histograms of Propidium iodide (PI) stained H9, FN2.1 and HF unfixed cells treated for 24 h with 5% O_2_, CoCl_2_ (250 µM) and DMOG (1 mM). Percentage of PI positive cells (late apoptotic or necrotic) was determined by flow cytometric analysis. Mean + SEM from three independent experiments are shown. Statistical analysis was done by Student’s t-test, (**) *p* < 0.01 and (***) *p* < 0.001 vs. Vehicle (DMSO).

We next wondered whether reduced levels of oxygen (5% O_2_) and chemical hypoxia induction affect hPSCs and human fibroblast (HF) viability. We included 5% O_2_ treatment as a direct comparator to the effects of DMOG or CoCl_2_ as long terms effects of cellular hypoxia (5% O_2_) proved to be beneficial to in vitro hESCs cell cultures. In addition, HF were used as an example of terminal differentiated cells and, particularly, the HF used were the ones from which hiPSCs line FN2.1 was reprogrammed. We determined the percentage of cell viability after 24 h incubation with 5% O_2_ and after 24 h of chemical hypoxia induction with increasing concentrations of CoCl_2_ and DMOG using a XTT/PMS vital dye assay. As shown in Fig. 1a, cell viability felt down significantly in H9 and FN2.1 cell lines with both compounds (CoCl_2_ and DMOG) while no significant changes were observed upon 5% O_2_ incubation. As expected, changes in cell viability were concentration dependent, and concentrations that reduced cell viability by 30–50% were chosen for further experiments (250 µM for CoCl_2_; 100 µM and 1 mM for DMOG). XTT assay is dependent on mitochondrial respiration, which is inhibited in a dose dependent manner by hypoxia-mimetic agents, for this reason we also measured cell viability by Trypan blue dye exclusion staining. Similar results were obtained when live and dead cells were counted using Trypan blue dye. As shown in Fig. 1b the percentage of surviving hPSCs (H9 and FN2.1) significantly decreased 24 h after CoCl_2_ (250 µM) and DMOG (1 mM) addition and no effect was found with 5% O_2_. As Trypan blue staining showed that DMOG 100 µM does not affect cell viability, 1 mM concentration was chosen for further experiments.

Additionally, we measured cell death by flow cytometry analysis of propidium iodide (PI) staining in H9, FN2.1 and HF cells. Histograms in Fig. 1c show the percentages of 5% O_2_^−^, CoCl_2_^−^, DMOG-treated or untreated cells exhibiting loss of plasma membrane integrity (late apoptosis or necrosis). We observed that only chemical hypoxia (CoCl_2_ 250 µM and DMOG 1 mM, 24 h treatments) induced apoptosis or necrosis in hPSCs. Importantly, HF resulted in less sensitive or not sensitive to the cytotoxicity triggered by CoCl_2_ and DMOG at the tested concentrations than hPSCs (Fig. 1b,c).

**Figure 2:**
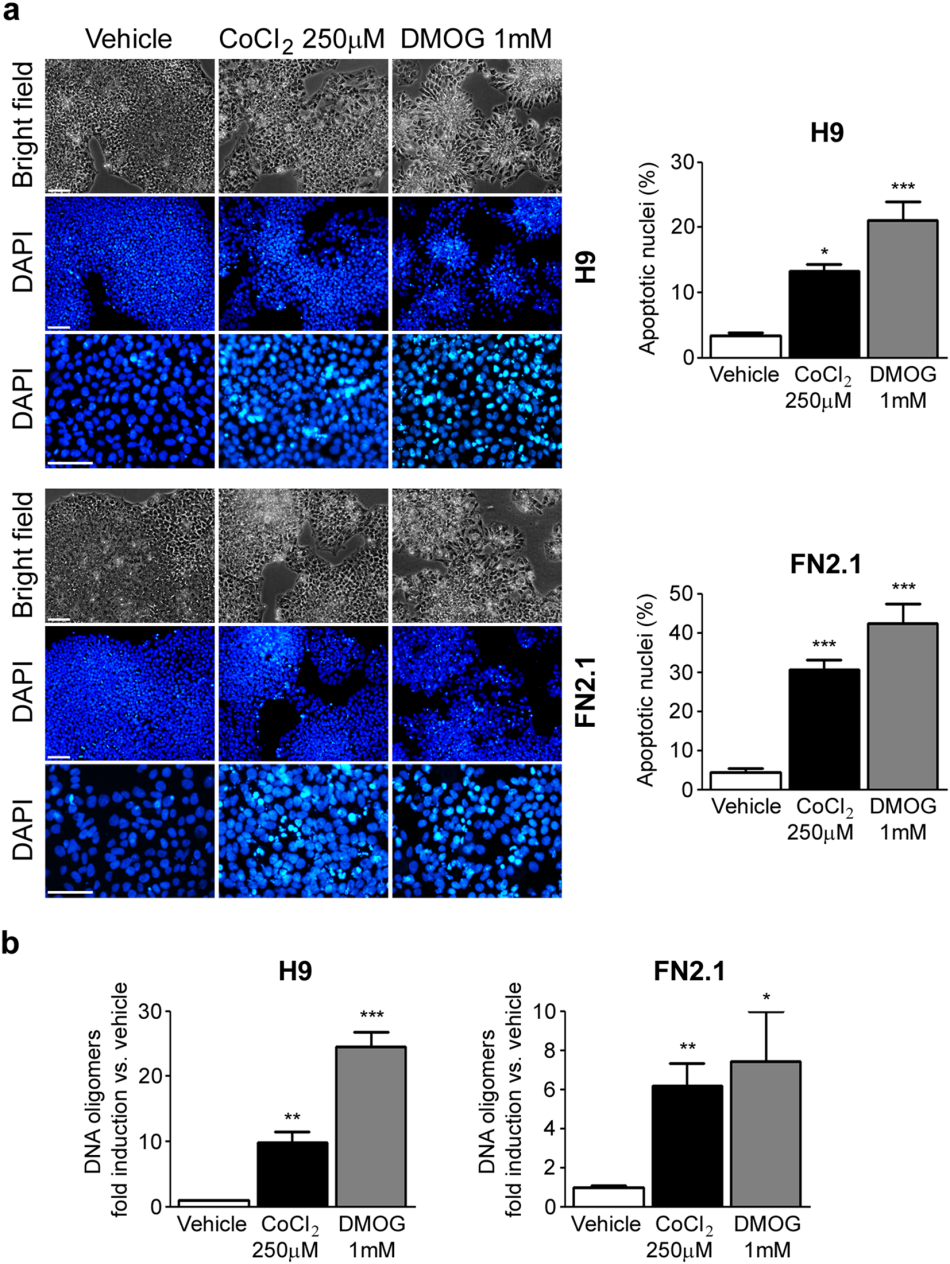
Chromatin condensation and DNA fragmentation upon CoCl_2_ and DMOG treatments. (**a**) Chromatin condensation was analyzed by DAPI staining 24 h after incubation of H9 and FN2.1 cells with CoCl_2_ (250 μM) and DMOG (1 mM). Figure shows representative images and means + SEM from at least five independent experiments are graphed for % of apoptotic nuclei. The scale bar represents 100 μm. Statistical analysis was done by one-way ANOVAs followed by Dunnett's multiple comparisons test, (*) *p* < 0.05 and (***) *p* < 0.001 vs. Vehicle (DMSO). (**b**) Genomic DNA fragmentation into oligomers of 180–200 bp or multiples of that was quantified in H9 and FN2.1 cells at 24 h post-chemical hypoxia induction with CoCl_2_ (250 μM) and DMOG (1 mM) treatments using a specific ELISA kit. Mean + SEM fold induction relative to Vehicle (DMSO) of at least three independent experiments are shown. Statistical analysis was done by Student’s t-test, (*) *p* < 0.05, (**) *p* < 0.01 and (***) *p* < 0.001 vs. Vehicle (DMSO).

Next, to elucidate whether the mode of cell death triggered by chemical hypoxia is via apoptosis we evaluated the appearance of apoptotic features in hESCs (H9) and hiPSCs (FN2.1). Importantly, as we observed no cell death induction, we stopped using 5% O_2_ treatment and HF as comparators. Chromatin condensation paralleled by ballooning and cell detachment are some of the criteria which are used to identify apoptotic cells. Therefore, we assessed these morphological changes by DAPI staining of nuclear DNA and bright-field microscopic images of cells subjected to chemical hypoxia or normoxia, respectively. We observed that chemical hypoxia (24 h treatment with CoCl_2_ 250 µM and DMOG 1 mM) increased both the percentage of hESCs and hiPSCs exhibiting intense bright DAPI staining, indicative of apoptotic nuclei, (3.4 ± 0.5% Vehicle, 13.2 ± 1.1% CoCl_2_ 250 µM and 21.1 ± 2.8% DMOG 1 mM for H9 cells; 4.5 ± 0.9% Vehicle, 30.6 ± 2.5% CoCl_2_ 250 µM and 42.5 ± 4.9% DMOG 1 mM for FN2.1 cells), and the presence of ballooned and detached cells (Fig. 2a).

We next measured DNA fragmentation (cytoplasmic oligonucleosomal fragments of approximately 180–200 bp, or multiples of that, representative of inter-nucleosomal cleavage of DNA), a late event in the apoptotic cascade. We quantified DNA oligomers with an immunoassay, using antibodies directed against DNA and histones. As shown in Fig. 2b, a significant increase in the proportion of DNA oligomers was observed 24 h after CoCl_2_ (250 µM) and DMOG (1 mM) treatments in both H9 and FN2.1 cells.

**Figure 3:**
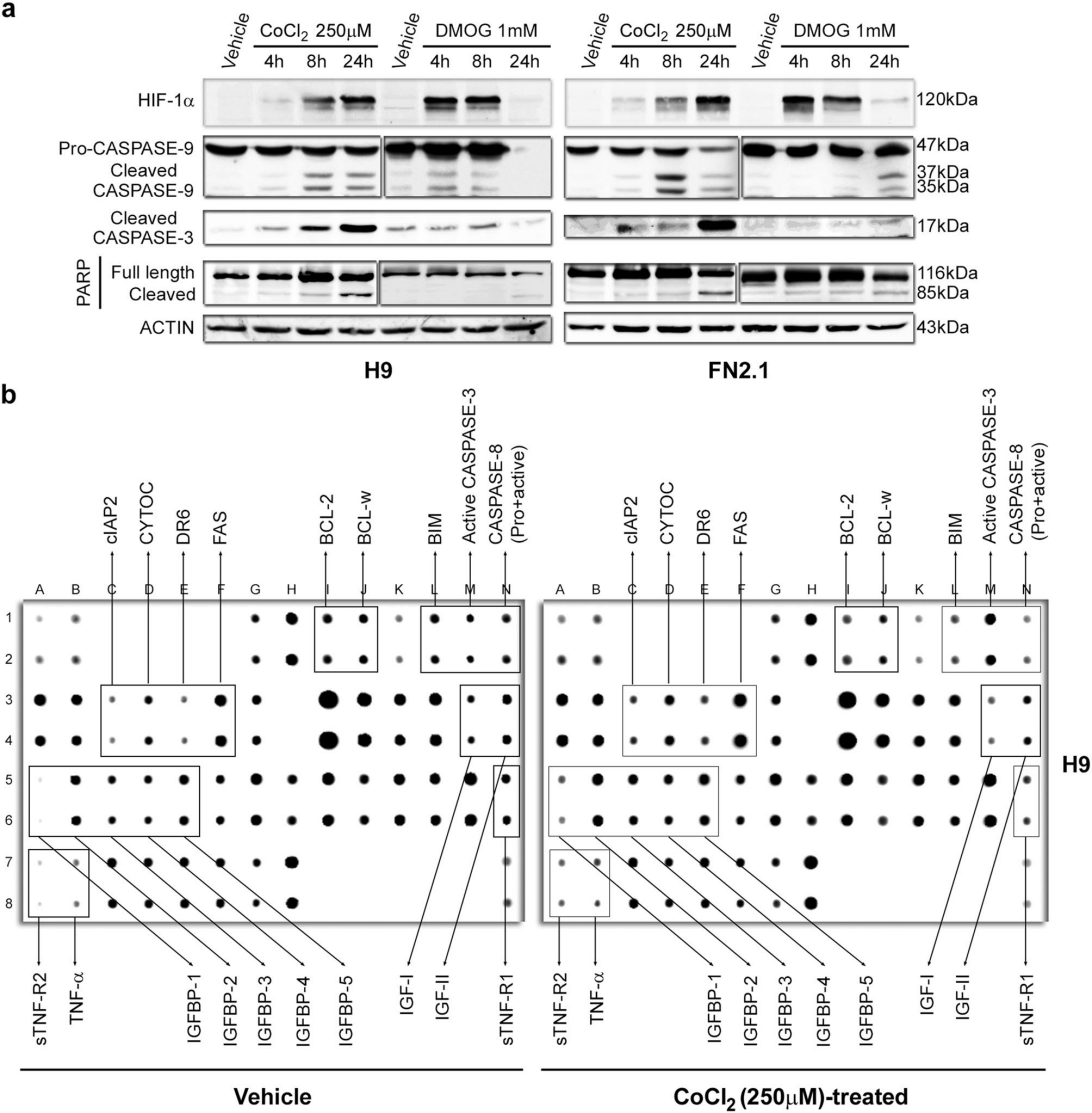
CASPASE-9, CASPASE-3 activation and PARP cleavage and apoptotic protein profiling upon chemical hypoxia induction. (**a**) Cleavage and activation of initiator CASPASE-9, effector CASPASE-3, PARP proteolysis (Caspase-3 substrate) and HIF-1α stabilization were analyzed by Western blot in H9 hESCs and FN2.1 hiPSCs at 4, 8- and 24-h post CoCl_2_ (250 µM) and DMOG (1 mM) treatments. ACTIN was used as loading control. Representative blots of three independent experiments are shown (full-length images are presented in Supplementary Fig. S10). Bar graphs representing densitometric quantification of bands are shown in Supplementary Fig. S3. (**b**) hPSCs (H9) were left untreated or treated with CoCl_2_ (250 µM) for 8 h. Equal total protein lysate was loaded on each Apoptosis Antibody array membrane. Antibody spots exhibiting signal differences are indicated in black boxes.

Activation of initiator and effector caspases is other relevant criteria to determinate apoptosis. Upon chemical hypoxia induction, initiator pro-CASPASE-9 (47 kDa) was cleaved into active fragments (37/35 kDa), as judged by the decrease in pro-CASPASE-9 and the increase in active fragments levels detected by Western blot in H9 and FN2.1 cells (Fig. 3a). Cleaved CASPASE-9 can further process other caspase members, including CASPASE-3, to initiate a caspase cascade, which leads to apoptosis. Western blot detection of cleaved CASPASE-3 (appearance of p17 fragment) revealed a time-dependent activation of CASPASE-3, which was accompanied by HIF-1α stabilization and CASPASE-9 activation, mediated by CoCl_2_ (250 µM) and DMOG (1 mM) treatments (Fig. 3a). Moreover, CASPASE-3 activation was also confirmed by immunofluorescence in both cell lines as soon as 6 h after chemical hypoxia induction (Supplementary Fig. S2). In parallel, time course studies showed the presence of cleaved PARP (effector caspases substrate) which was preceded by the appearance of the catalytically active fragment p17. This chronology is compatible with the involvement of CASPASE-3 in PARP proteolysis (Fig. 3a and Supplementary Fig. S3). Importantly, we determined that chemical hypoxia induction resulted in the activation of apoptotic caspases with very similar kinetics in hiPSCs FN2.1 and hESCs H9. Interestingly, kinetics of HIF-1α stabilization was accelerated in both FN2.1 and H9 cells when DMOG was used at the tested concentration (Fig. 3a and Supplementary Fig. S3).

As a whole, the above results indicate that chemical hypoxia induces cell death mainly via apoptosis in hPSCs. Besides, based on the presence of cleaved CASPASE-9, the mitochondrial-mediated apoptosis pathway participates in this event.

### Apoptotic protein profiling in chemical hypoxia-treated hPSCs

In order to gain further insights about the pathways and potential mechanisms by which chemical hypoxia may regulate apoptosis in hPSCs, we performed an exploratory experiment by screening 43 human apoptosis-related proteins using a human Apoptosis Antibody Array. In fact, H9 hESCs treated with CoCl_2_ (250 µM) for 8 h showed a changed expression profile of apoptosis-related proteins (Fig. 3b). Importantly, similarly as previously reported in Fig. 3a and Supplementary Fig. S2, we observed an enhanced active CASPASE-3 immunoreactivity in CoCl_2_-treated cells compared to Vehicle (H_2_O)-treated counterparts (Fig. 3b and Supplementary Fig. S4). Intriguingly, it came to our attention a marked reduction in CASPASE-8 signal that could be a cause of caspase proteolysis and activation due to extrinsic apoptosis pathway induction. This hypothesis was further reinforced by the increased expression levels observed of several apoptosis-related proteins involved in the extrinsic pathway, like TNF-α, sTNF-R2, IGFBP family, FAS and Death receptor 6 (DR6) (Fig. 3b and Supplementary Fig. S4). Moreover, in what respect to the mitochondria-mediated apoptosis pathway, changes in CYTOCHROME C and c-IAP-2 (both upregulated), as well as IGF-I, IGF-II, BIM, BCL-2 and BCL-w (all downregulated) were detected (Fig. 3b and Supplementary Fig. S4). Also, worth mentioning the strong downregulation of IGF-I and IGF-II, cytokines that by activating ERK1/2 and PI3K/AKT pathways turn out to be essential for hPSCs self-renewal and viability^16–18^.

**Figure 4:**
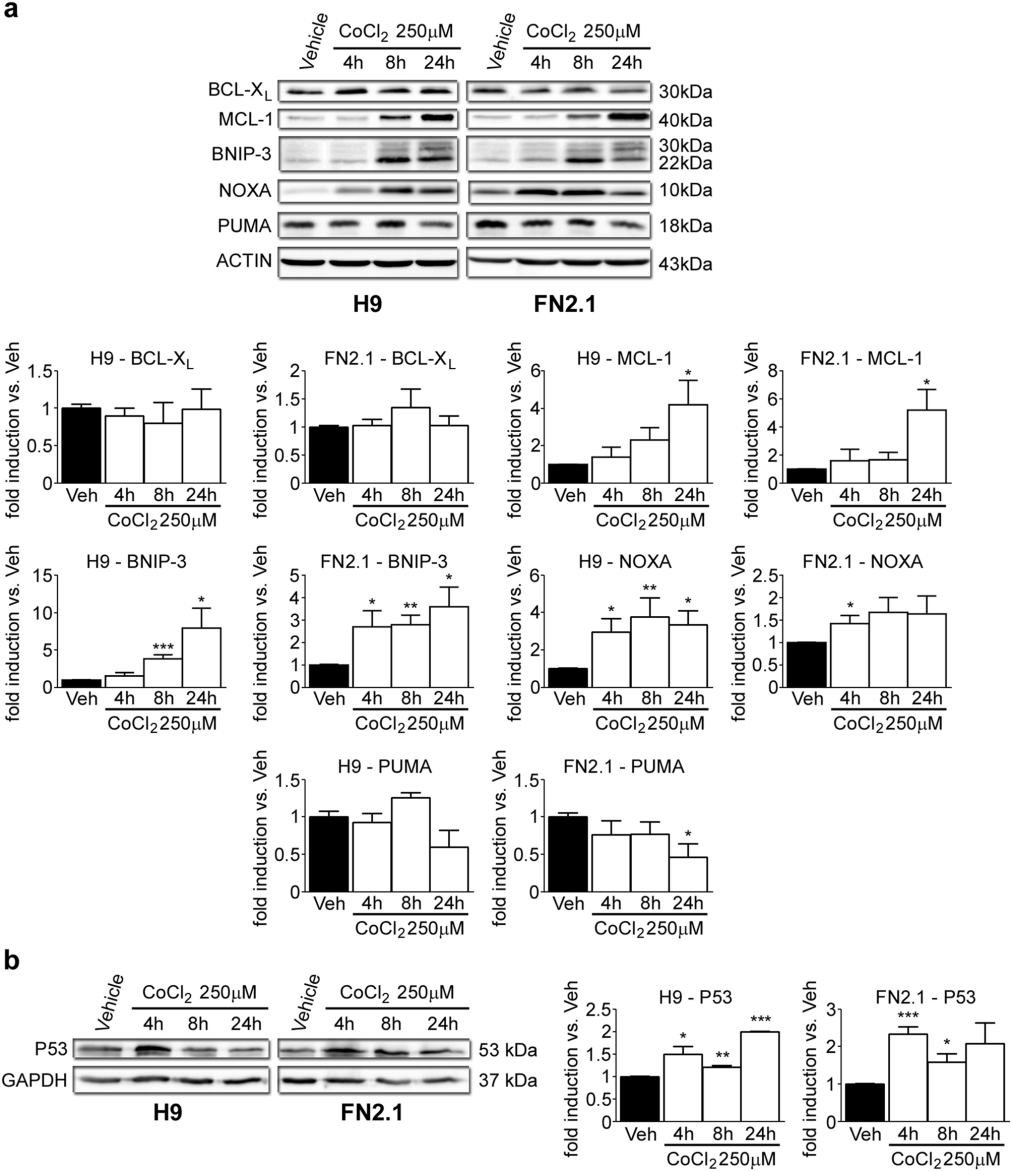
BCL-2 family members and p53 expression levels. Expression levels of (**a**) BCL-2 family members, including BCL-X_L_ (anti-apoptotic), MCL-1 (anti-apoptotic), BNIP-3 (pro-apoptotic), NOXA (pro-apoptotic) and PUMA (pro-apoptotic) or (**b**) P53 were analyzed by Western blot in H9 and FN2.1 cells at 4, 8 and 24 h post CoCl_2_ (250 µM) treatment. ACTIN or GAPDH were used as loading control. Representative blots of three independent experiments are shown (full-length images are presented in Supplementary Fig. S11). Bar graphs represent densitometric quantification of bands. Data are expressed as means + SEM fold induction relative to Vehicle (H_2_O) (arbitrarily set as 1) and Statistical analysis was done by Student’s t-test, (*) *p* < 0.05, (**) *p* < 0.01 and (***) *p* < 0.001 vs. Vehicle (H_2_O).

hPSCs are very susceptible to undergo apoptosis due to their higher state of mitochondrial priming, a lowered cell intrinsic threshold for initiating mitochondrial induced apoptosis, based on the balance of pro- and anti-apoptotic protein members of the BCL-2 family^19^. Bearing this in mind, we next quantified key BCL-2 family members in hPSCs (not present in the Apoptosis Array) expression levels after chemical hypoxia induction. Particularly BCL-X_L_, a key anti-apoptotic protein; BNIP-3, a well-known pro-apoptotic protein which is induced upon hypoxic stimuli^20^; MCL-1 (anti-apoptotic), PUMA (pro-apoptotic) and NOXA (pro-apoptotic) which are highly expressed and rapidly responding proteins in hPSCs^21,22^. To address this issue, Western blot assays were performed to analyze expression levels of these BCL-2 family members in hPSCs at 4, 8 and 24 h post-chemical apoptosis induction with CoCl_2_ (250 µM) treatment. Results shown in Fig. 4a indicate that BNIP-3, MCL-1 and NOXA expression levels were significantly up-regulated at different time points upon chemical hypoxia induction. On the contrary, PUMA expression levels decreased at 24 h post-CoCl_2_ treatment. Moreover, these increments were also observed in *BNIP-3*, *BNIP-3L* (also known as *NIX*, which is a *BNIP-3* homologue), *MCL-1* and *NOXA* mRNA expression levels quantified by RT-qPCR (Supplementary Figs. S1 and S5).

P53 is a well characterized tumor suppressor transcription factor that gets activated by cellular stress events such as DNA damage and hypoxia, among others^23^. One of P53 functions is to induce transcription of genes that code for some proteins involved in the intrinsic pathway of apoptosis such as *PUMA*, *NOXA* and *BAX*^24^. In order to study the possible participation of P53 in our chemical model of hypoxia we first measured P53 levels by Western blot in H9 and FN2.1 cells treated with CoCl_2_ (250 µM) for 4, 8 and 24 h. Interestingly, we found out that P53 expression levels were significantly up-regulated at different time points in both cell lines (Fig. 4b). In consequence, P53 may participate in chemical hypoxia-induced apoptosis in hPSCs.

### Involvement of HIF-1α and HIF-2α in chemical hypoxia-induced apoptosis in hPSCs.

**Figure 5:**
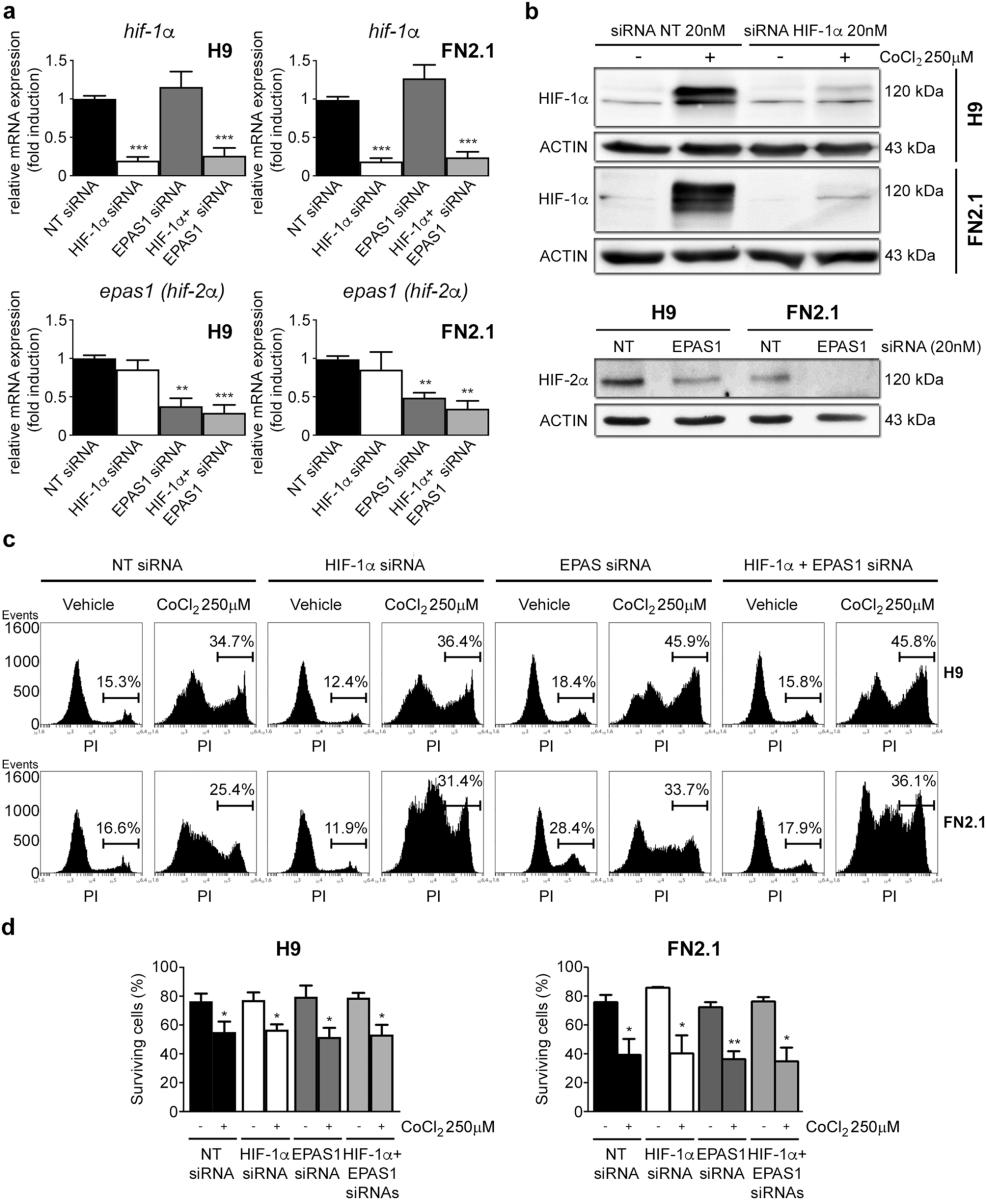
Effect of siRNA-mediated down regulation of HIF-1α and HIF-2α in hPSCs cell viability and death upon chemical hypoxia induction. H9 hESCs and FN2.1 hiPSCs were transfected with negative control non-targeting siRNA (NT siRNA) (20 nM), HIF-1α siRNA (20 nM) and EPAS1 (HIF-2α) siRNA (20 nM) and then: (**a**) mRNA expression levels of *HIF-1α* and *EPAS1* (*HIF-2α*) were analyzed by RT-qPCR at 48 h post siRNAs transfection. *RPL7* mRNA expression levels were used as normalizer. Graph shows mean + SEM mRNA fold induction relative to NT siRNA transfectants arbitrarily set as 1 from three independent experiments. Statistical analysis was done by Student’s t-test, (**) *p* < 0.01 and (***) *p* < 0.001 vs. NT siRNA. (**b**) Expression levels of HIF-1α and HIF-2α were analyzed by Western blot in H9 and FN2.1 cells at 48 h post siRNAs transfection. HIF-1α was stabilized with CoCl_2_ (250 µM for 24 h) treatment. ACTIN was used as loading control. Representative blots are shown (full-length images are presented in Supplementary Fig. S12). (**c**) Representative histograms PI stained H9 and FN2.1 unfixed cells at 48 h post siRNA transfection. Chemical hypoxia was induced with CoCl_2_ (250 µM) at 24 h post siRNA transfection. Percentage of PI positive cells (late apoptotic or necrotic) was determined by flow cytometric analysis. Vehicle: H_2_O. Graph and statistically analysis from three independent experiments are shown in Supplementary Fig. S6. (**d**) Histograms show percentage of surviving cells assessed by Trypan blue exclusion method at 48 h post siRNA transfection. 24 h after transfection cells were treated with CoCl_2_ (250 µM). Mean + SEM from at least three independent experiments are shown. Statistical analysis was done by Student’s t-test, (*) *p* < 0.05 and (**) *p* < 0.01 vs. NT siRNA.

Next, to test HIF-1α and HIF-2α involvement in chemical hypoxia-induced apoptosis in hPSCs, we used siRNA to downregulate both HIFs expression. The efficiency of siRNA- knockdown was monitored by RT-qPCR and Western blot in hESCs (H9) and hiPSCs (FN2.1) cultured in defined E8 medium and transfected with either non-targeting control siRNA (NT-siRNA) or specific siRNA. As shown in Fig. 5a,b, siRNA transfection led to a significant decrease in *HIF-1*α and *HIF-2*α mRNA and protein expression levels (in the case of *HIF-1*α*,* protein expression levels were knocked-down even in CoCl_2_-treated cells). Interestingly, we found that in hPSCs siRNA-mediated downregulation of both HIF-1α and HIF-2α was not able to revert the increased apoptosis or necrosis induced by chemical hypoxia (CoCl_2_ 250 µM and DMOG 1 mM) as judged by PI staining and Trypan blue dye exclusion data (Fig. 5c,d and Supplementary Figs. S6 and S7). Taken together, the above results suggest that chemical hypoxia induces hPSCs cell death by a HIF-1α and HIF-2α independent mechanism. In this sense, we observed a strong inhibition on the induction of *BNIP-3* and *BNIP-3L* mRNA, and BNIP-3 protein expression levels induced by CoCl_2_ treatment in HIF-1α siRNA transfected hPSCs (Supplementary Fig. S8a,b). These last results suggest that these pro-apoptotic factors are not involved in chemical hypoxia apoptosis induction. Importantly, we found that in hPSCs an efficient siRNA-mediated downregulation of BNIP-3 was not able to revert the increased apoptosis or necrosis induced by chemical hypoxia (CoCl_2_ 250 µM) as judged by PI staining and Trypan blue dye exclusion data confirming the above finding (Supplementary Fig. S8c, d and e).

### Participation of P53 and NOXA on apoptosis triggered by chemical hypoxia

**Figure 6:**
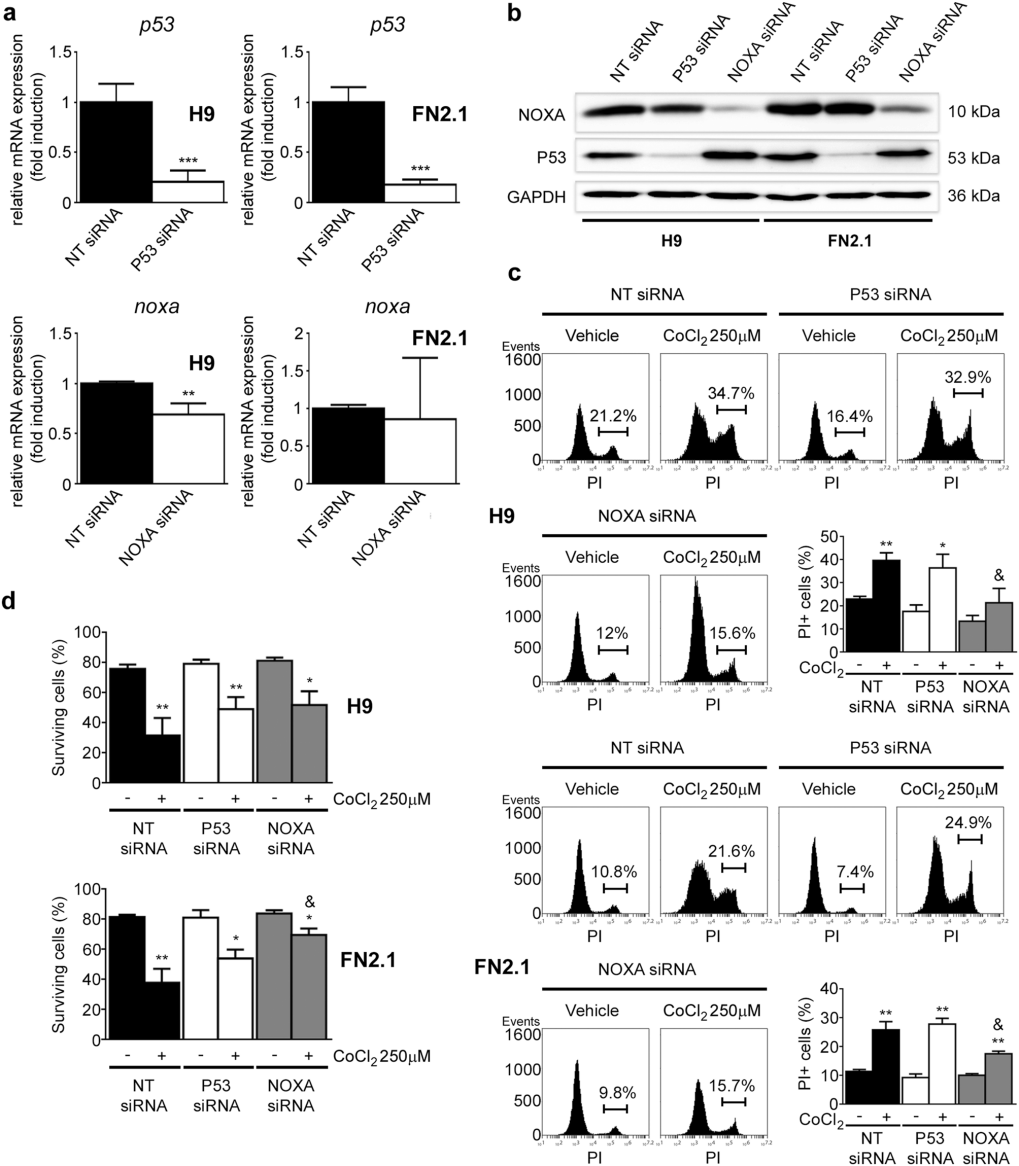
Effect of siRNA-mediated down regulation of P53 and NOXA in hPSCs cell viability and death upon chemical hypoxia induction. H9 hESCs and FN2.1 hiPSCs were transfected with negative control non-targeting siRNA (NT siRNA) (20 nM) or P53 siRNA (20 nM) or NOXA siRNA (20 nM) and then: (**a**) mRNA expression levels of *P53* and *NOXA* were analyzed by RT-qPCR at 48 h post siRNAs transfection. *RPL7* mRNA expression levels were used as normalizer. Graph shows mean + SEM mRNA fold induction relative to NT siRNA transfectants arbitrarily set as 1 from three independent experiments. Statistical analysis was done by Student’s t-test, (**) *p* < 0.01 and (***) *p* < 0.001 vs. NT siRNA. (**b**) Expression levels of NOXA and P53 were analyzed by Western blot in H9 and FN2.1 cells at 48 h post siRNAs transfection. GAPDH was used as loading control. Representative blots of three independent experiments are shown (full-length images are presented in Supplementary Fig. S13). (**c**) Representative histograms of PI stained H9 and FN2.1 unfixed cells at 48 h post siRNA transfection. Chemical hypoxia was induced with CoCl_2_ (250 µM) at 24 h post siRNA transfection. Percentage of PI positive cells (late apoptotic or necrotic) was determined by flow cytometric analysis. Vehicle: H_2_O. Mean + SEM from three independent experiments are shown. Statistical analysis was done by Student’s t-test, (*) *p* < 0.05 and (**) *p* < 0.01 vs. NT siRNA; (&) *p* < 0.05 vs. NT + CoCl_2_ 250 µM. (**d**) Bar graphs show percentage of surviving cells assessed by Trypan blue exclusion method at 48 h post siRNA transfection. 24 h after transfection cells were treated with CoCl_2_ (250 µM). Mean + SEM from at least three independent experiments are shown. Statistical analysis was done by Student’s t-test, (*) *p* < 0.05 and (**) *p* < 0.01 vs. NT siRNA; (&) *p* < 0.05 vs. NT + CoCl_2_ 250 µM.

Later, as P53 and NOXA were up-regulated upon CoCl_2_ (250 µM) treatment in hPSCs, we aimed to study the participation of these two potential pro-apoptotic proteins in hPSCs CoCl_2_-induced apoptosis regulation by using a siRNA approach. Again, the efficiency of each siRNA- knockdown was first monitored by RT-qPCR and Western blot in H9 hESCs and FN2.1 hiPSCs cultured in defined E8 medium and transfected with either NT-siRNA or specific siRNAs. As shown in Fig. 6a,b, siRNA transfection led to a significant decrease in *P53* mRNA and protein expression levels. However, in the case of *NOXA*, although only a slight downregulation in mRNA expression levels was observed, a strong silencing of its protein levels was found. It could be the case that, as *NOXA* is a rapid response gene, its fast transcription compensates siRNA-mediated *NOXA* mRNA downregulation.

Interestingly, we found that in hPSCs siRNA-mediated downregulation of NOXA but not P53 was able to at least partially revert the increased apoptosis or necrosis induced by chemical hypoxia (CoCl_2_ 250 µM) as judged by PI staining and Trypan blue dye exclusion data (Fig. 6c,d). Notably, on the case of P53, a partial but not significant reversion was observed only on the Trypan blue dye exclusion experiments (Fig. 6d).

Finally, we wondered if a similar mechanism was involved in DMOG-induced apoptosis of hPSCs. Similar to what we observed with CoCl_2_-treatment, P53 and NOXA were up-regulated upon DMOG (1 mM) treatment in hPSCs (Supplementary Fig. S9a). Again, siRNA-mediated downregulation of NOXA but not P53 was able to at least partially revert the increased apoptosis or necrosis induced by chemical hypoxia (DMOG 1 mM) in hPSCs as judged by PI staining and Trypan blue dye exclusion data (Supplementary Fig. S9b,c).

Taken together, the above results suggest that NOXA mediates, at least in part, cell death induction by chemical hypoxia in hPSCs.

## Discussion

hPSCs, as previously mentioned, hold great potential to provide cellular therapy for a range of diseases, being also invaluable sources for the study of human development and disease, as well as for drug discovery. For this challenge to be achieved with safety and efficiency, hPSCs need to be maintained in vitro as highly pluripotent populations in the absence of spontaneous differentiation and with a tight control of cell viability. Accumulating data suggests that environmental in vitro cell culture conditions and specifically O_2_ tension have an impact on the maintenance of hPSCs proliferation, self-renewal and stemness. Use of low environmental O_2_ tensions (for example 5%) has been shown to promote self-renewal, to increase expression of key pluripotency markers, to reduce the amount of spontaneous differentiation and to decrease incidence of chromosomal abnormalities and cell death rate^9–11,25^.

Key regulators of the hypoxia response are HIF-1α and HIF-2α. These transcription factors exert a complex role regulating more than 100 genes including glycolytic enzymes and survival factors, which are required to cope with low oxygen tensions. Herein, we explored the effect of stabilizing HIF-1α and HIF-2α with two structurally independent prolyl-hydroxylase inhibitors CoCl_2_ and DMOG in hPSCs under ambient air. We found that both agents were effective in stabilizing HIF-1α in hESCs and hiPSCs lines. However, in the case of HIF-2α we observed that basal expression levels were not induced upon chemical hypoxia induction. Moreover, CoCl_2_ and DMOG treatments markedly reduced cell viability as well as induced typical cell death and apoptotic features, like cell shrinkage, ballooning and detachment, chromatin condensation and nuclear fragmentation.

Furthermore, in the present study, we observed an intricate balance between factors that induce or counteract apoptosis during chemical hypoxia stimulation, which finally ended in programmed cell death induction. The main mechanisms of apoptosis are the intrinsic (mitochondrial mediated) and the extrinsic (death receptor mediated) pathways. It has become clear that intrinsic and extrinsic pathways are not mutually exclusive and components of both may co-participate in the regulation of the apoptotic program in response to a single stimulus. Moreover, effector caspases (like CASPASE-3) are key and common executioner proteases in both apoptosis-induction pathways^26^. In this sense, we showed that CASPASE-3 activity was enhanced in CoCl_2_- and DMOG-treated hPSCs and may play a key role in chemical induced-apoptosis, which is consistent with previous studies in mESCs and PC12 cells^12,27^. Besides, based on CASPASE-9 (initiator caspase of the intrinsic pathway) cleavage and activation, we found that intrinsic pathway participates in chemical hypoxia induced-apoptosis in hPSCs. Even more, by an exploratory screening of 43 human apoptosis-related proteins, we also observed changes in extrinsic pathway related proteins (CASPASE-8, TNF-α, sTNF-R2, IGFBP family, FAS and DR6) that indicates that the death receptor mediated apoptosis pathway might also participate in chemical hypoxia induced-apoptosis in hPSCs. Nevertheless, more experiments should be performed in order to confirm this hypothesis. Interestingly, Lee, J. H. et al*.* achieved similar conclusions in mESCs upon CoCl_2_ treatment^12,28^.

HIF-1α, depending on the type and status of the cells and the severity of oxygen deprivation, can prevent cell death, induce apoptosis or even stimulate cell proliferation^29^. In solid tumors, as an example, severe hypoxia in the presence of energy stimulates cells to undergo apoptosis, whereas oxygen levels above 0.5% prevent cell death^29^. This could also be the case in hPSCs, which share the predominance of glycolytic metabolism with cancer cells^30^.

Hypoxia induces apoptosis at least in two ways: by HIF-1α stabilization and by ROS generation^29^. In fact, HIF-1α, acting in combination with other factors, can either induce or inhibit apoptosis^31,32^.

Both HIF-1α and HIF-2α play critical roles in regulating responses of different cell types exposed to hypoxic environments. Thus, we wondered whether HIF-1α and HIF-2α were also key mediators of chemical hypoxia-induced apoptosis in hPSCs. HIF-1α can induce apoptosis by P53 stabilization^33^ or by transcriptional upregulation of pro-apoptotic proteins like BNIP-3 and BNIP-3L^15,34,35^. However, until the present, the complicated hypoxic cell death pathways cannot be explained only by the induction of these few well-known pro-apoptotic responsive genes. In fact, herein we demonstrated that chemical hypoxia induced *P53* (protein levels), *BNIP-3* (mRNA and protein levels) and *BNIP-3L* (mRNA levels). Even though, we found that siRNA-mediated downregulation of HIF-1α and HIF-2α did not significantly affect the extent of cell death triggered by CoCl_2_ and DMOG in hPSCs, suggesting the participation of alternative pathways. In line with this finding, we demonstrated that siRNA-mediated BNIP-3 gene silencing was also not able to revert apoptosis induction upon CoCl_2_ treatment. In consequence, these proteins probably are not critical mediators of chemical hypoxia-induced apoptosis in hPSCs. Interestingly, others achieved similar conclusions in a different cellular context^36^.

As previously mentioned, P53 is a transcription factor that can promote cell survival and adaptation to mild stress as well as induce cell death upon confrontation with severe stress. Importantly, P53 activation by chemical hypoxia was found to be a HIF-1α independent mechanism of apoptosis induction in human lung cells^37^. In fact, to gain insight into this possible mechanism of chemical hypoxia-induced cell death regulation in hPSCs, we use siRNA methods to reduce P53 expression levels. However, despite the fact that P53 was upregulated in hPSCs exposed to chemical hypoxia its downregulation did not significantly affect the rate of cell death observed in NT-siRNA transfectants, indicating that this transcription factor is not a key player in chemical hypoxia-induced apoptosis in hPSCs.

NOXA, a BH3-only mitochondrial protein that contributes to apoptosis by disrupting mitochondrial outer membrane integrity, is a mediator of P53- and HIF-1α-dependent or -independent cell death that can be transcriptionally activated under hypoxic environments^38–41^. Besides, NOXA is highly expressed in hPSCs^21^ and is responsible for the highly sensitive mitochondrial apoptosis observed in the aforementioned stem cells^42^. The fact that NOXA was upregulated after CoCl_2_ and DMOG treatment prompted us to explore its role in the regulation of hPSCs death. To do so, we transfected cells with specific siRNA and found that diminished NOXA levels protected cells from CoCl_2_ or DMOG induced apoptosis. In this sense, it is well known that CoCl_2_, can induce cell death through oxidative stress by increasing ROS generation^43,44^. Recently, it has been reported that the generation of ROS at the endoplasmic reticulum leads to the upregulation of NOXA, through activation of the ATF4 (activating transcription factor 4)/CHOP (C/EBP-homologous protein) axis, and culminates in cell death^45–47^. Thus, considering that neither the downregulation of HIF-1α or HIF-2α nor the one of P53 protected hPSCs from chemical hypoxia-induced cell death, the upregulation of NOXA by ATF4/CHOP cascade, although unproven, could be an alternative pathway that regulates hPSCs fate upon CoCl_2_ or DMOG exposure (chemical hypoxia and ROS generation).

Finally, therapeutic application of hPSCs-differentiated progenies carries the potential to form teratomas (composed from cells of the three embryonic germ layers), due to the presence of residual undifferentiated cells. Hence, the removal of these contaminating undifferentiated hPSCs has been considered a critical requirement for hPSCs-based clinical applications^48^. As an example, etoposide treatment of early cardiac progenitors derived from hiPSCs was shown to be effective in eliminating residual hiPSCs thus decreasing the effect of teratoma formation^49^. In this sense, chemical hypoxia induction, particularly CoCl_2_ or DMOG treatments, might be a suitable treatment to eliminate potentially teratogenic hPSCs contamination from more resistant differentiated derivatives thus ensuring safer clinical applications. However, it would only be useful on more ROS-resistant or antioxidant-rich differentiated progenies (as human fibroblasts, HF) and right now is only a speculation that requires further investigation.

## Methods

### Cell lines and culture

hESCs line WA09 (H9)^1^ was purchased from WICELL RESEARCH INSTITUTE (http://www.wicell.org) at low passages (p15 to p20). hiPSCs line FN2.1 were derived from human foreskin fibroblasts (HF) at our laboratory per under relevant guidelines and regulations and has been fully validated^50^. All experimental protocols where hiPSCs FN2.1 line was used, including derivation, were given ethical approval by the local Ethics Committee (Comité de ética en investigaciones biomédicas del Instituto FLENI) and written informed consent was obtained from donor prior to foreskin fibroblasts isolation. hPSCs were maintained on Vitronectin (0.5 µg/cm^2^) coated dishes (VTN-N, GIBCO) in combination with fully defined Essential 8 medium (E8, GIBCO). Cultures were split every 3 to 4 days by means of PBS-EDTA (Versene, GIBCO) passaging. Before experiments, hPSCs grown on Vitronectin and E8 were dissociated into single cells using Accutase 1x (GIBCO) for 7 min, plated onto Vitronectin coated dishes (with addition of 10 µM Y-27632 ROCK inhibitor) and grown until confluence with E8. Human foreskin fibroblasts (HF) were prepared as primary cultures from freshly obtained human foreskins as previously described^51^ after obtaining written informed consent from donor according to guidelines established by the Ethics Committee of FLENI. All cell lines were free of *Mycoplasma* *sp.* infection, which was tested as previously described^52^.

### Reagents and hypoxia induction

Cobalt (II) chloride hexahydrate (CoCl_2_) (SIGMA, C8661) and dimethyloxalylglycine (DMOG) (CALBIOCHEM, 400091) were dissolved in H_2_O and DMSO, respectively, and stored at − 80 °C protected from light. Reagents were added to cell cultures such that the final concentrations were not higher than 0.10% (v/v). Both CoCl_2_ and DMOG were used to induce chemical hypoxia in hPSCs. Particularly, CoCl_2_ is a chelating agent that displace Fe^2+^ from the active center of prolyl-hydroxylases, inhibiting them and preventing Hypoxia-inducible transcription factor 1α (HIF-1α) degradation^13^. DMOG also affects the HIF prolyl-hydroxylases, inhibiting them competitively^14^. The result in both cases is the increase in HIF-1α intracellular levels. Furthermore, 5% O_2_ cellular hypoxia was achieved using a modular hypoxia incubator chamber (Galaxy CO-14S, EPPENDORF NEW BRUNSWICK).

### RNA isolation and RT-qPCR

Total RNA was extracted from hPSCs with TRIzol (THERMO SCIENTIFIC) and cDNA was synthesized from 500 ng of total RNA with 15 mM of random hexamers and MMLV reverse transcriptase (PROMEGA), according to manufacturer's instructions and as previously described^18,51^. For qPCR studies, cDNA samples were diluted fivefold and PCR amplification and analysis were performed with STEPONEPLUS REAL TIME PCR SYSTEM (PE APPLIED BIOSYSTEMS). The FASTSTART UNIVERSAL SYBR GREEN MASTER MIX (ROX) (ROCHE) was used for all reactions, following manufacturer´s instructions. For information about primers sequences please see Supplementary methods (Table S1).

### Protein analysis

Protein expression levels were analyzed as previously described^18,51,53^. Briefly, total proteins were extracted from hPSCs in ice-cold RIPA protein extraction buffer (SIGMA) supplemented with protease inhibitors. Protein concentration was determined using BICINCHONINIC ACID PROTEIN ASSAY (PIERCE). Equal amounts of protein were electrophoresed on a 12% SDS-polyacrylamide gel and transferred to PVDF membranes. Blots were blocked 1 h at RT (room temperature) in TBS (20 mM Tris-HCl, pH 7.5, 500 mM NaCl) containing low-fat powdered milk (5%) and Tween 20 (0.1%). Incubations with primary antibodies were performed ON (overnight) at 4 °C in blocking buffer (3% skim milk, 0.1% Tween, in Tris-buffered saline). Membranes were then incubated with the corresponding counter-antibody and the proteins revealed by enhanced chemiluminescence detection (SUPERSIGNAL WEST FEMTO SYSTEM, THERMO SCIENTIFIC). For information about antibodies used please see Supplementary methods (Table S2). Densitometric analysis of protein levels were performed with IMAGEJ 1.34S SOFTWARE (WAYNE RASBAND, NATIONAL INSTITUTES OF HEALTH, https://imagej.nih.gov/ij/).

### Apoptosis protein antibody array membrane analysis

Relative levels of 43 human apoptosis-related proteins (Array Map information available in Supplementary methods, Table S3) were detected and analyzed using the human APOPTOSIS ARRAY KIT (ab134001, ABCAM), following manufacturer´s instructions. Briefly, membranes containing immobilized apoptosis-related antibodies were blocked for 2 h with BSA (bovine serum albumin for) on a rocking platform at RT. Membranes were then incubated with lysates of untreated or CoCl_2_ (250 µM)-treated H9 hESCs, along with Detection Antibody Cocktail (biotin-conjugated antibodies) ON at 4 °C. Finally, membranes were incubated with Streptavidin horseradish peroxidase conjugates and revealed by chemiluminescence detection. Densitometric analysis of protein levels were performed with IMAGEJ 1.34S SOFTWARE and values normalized against positive control spots following manufacturer´s instructions.

### Cell viability assay

hPSCs were plated onto Vitronectin coated 96-well dishes at densities between 1 × 10^4^–3 × 10^4^ cells per well and grown until confluence. 24 h post-treatments, 50 μg/well of activated 2,3-bis (2-methoxy-4-nitro-5-sulfophenyl)-5 [(phenylamino) carbonyl]-2 H-tetrazolium hydroxide (XTT) in PBS containing 0.3 μg/well of N-methyl dibenzopyrazine methyl sulfate (PMS) were added (final volume 100 μl) and incubated for 1–2 h at 37 °C. Cellular metabolic activity was determined spectrophotometrically at 450 nm^18,53^.

### Trypan blue staining

For Trypan blue exclusion assay, hPSCs were seeded on Vitronectin coated 6-well tissue culture plates at a density of 1 × 10^5^ cells/ml. At 24 h post-treatments, adherent and detached cells were collected and stained with 0.4% Trypan blue solution (final concentration 0.08%) for 5 min at room temperature. Cells were counted in a hemocytometer chamber. Percentages of surviving cells (unstained) were calculated as total number of live cells divided by total number of cells (stained) and multiplied by 100^18,53^.

### Flow cytometric analysis of cell viability by Propidium Iodide (PI) staining

Cell viability was analyzed by PI staining as previously described^18,53^. Briefly, 24 h after treatments, single-cell suspensions were obtained with Accutase (37 °C for 7 min). hPSCs were then centrifuged at 200 × *g* for 5 min and resuspended up to 1 × 10^6^ cells/ml in FACS Buffer (2.5 mM CaCl_2_, 140 mM NaCl and 10 mM HEPES pH 7.4). Next, 100 µl of cellular suspension were incubated with 5 µl of PI (50 µg/ml) in PBS for 5 min in the dark. Finally, 400 µl of FACS Buffer were added to each tube and cells were immediately analyzed by flow cytometry. Data was acquired on a BD ACCURI C6 FLOW CYTOMETER and analyzed using BD ACCURI C6 SOFTWARE.

### DAPI staining

hPSCs were grown on Vitronectin coated MWx24 cell culture dishes with E8 medium and, 24 h post-treatments, rinsed with ice-cold PBS and fixed in PBSA (PBS with 0.1% bovine serum albumin) with 4% formaldehyde for 45 min. After two washes cells were permeabilized with 0.1% Triton X-100 in PBSA with 10% normal goat serum for 30 min, washed twice and stained with stained with 4-6-Diamidino-2-phenylindole (DAPI) for 20 min. Stained cells were examined under a NIKON ECLIPSE TE2000-S inverted microscope equipped with a 20X E-Plan objective and a super high-pressure mercury lamp. The images were acquired with a NIKON DXN1200F digital camera, which was controlled by the ECLIPSENET SOFTWARE (version 1.20.0 build 61)^51^. Percentages of apoptotic nuclei were calculated as total number of cells showing chromatin condensation divided by total number of cells and multiplied by 100.

### Assessment of DNA fragmentation

Apoptosis induction was quantified by direct determination of nucleosomal DNA fragmentation with CELL DEATH DETECTION ELISAPLUS KIT (ROCHE) as previously described^18^. Briefly, 2 × 10^5^ hPSCs were plated on 24-well culture plates in 500 μl cell culture media. 24 h after chemical hypoxia induction, cells were lysed according to manufacturer's instructions, followed by centrifugation (200 × *g*, 5 min). The mono and oligonucleosomes in the supernatants were determined using an anti-histone-biotinylated antibody. The resulting color development was measured at 405 nm wavelength using a multiplate spectrophotometer. Results were expressed as DNA oligomer fold induction versus vehicle (DMSO), calculated from the ratio of absorbance of treated samples to that of untreated ones.

### Immunostaining and fluorescence microscopy

hPSCs were analyzed for in situ immunofluorescence as previously described^51,53^. Briefly, cells were rinsed with ice-cold PBS and fixed in PBSA (PBS with 0.1% bovine serum albumin) with 4% formaldehyde for 45 min. After two washes cells were permeabilized with 0.1% Triton X-100 in PBSA with 10% normal goat serum for 30 min, washed twice and stained with a rabbit polyclonal antibody anti-active CASPASE-3 (ab13847, ABCAM). Fluorescent secondary antibody Alexa Fluor 488-conjugated anti-rabbit IgG was purchased from Invitrogen and was used to localize the antigen/primary antibody complexes. Cells were counterstained with DAPI and examined under a NIKON ECLIPSE TE2000-S inverted microscope equipped with a 20X E-Plan objective and a super high-pressure mercury lamp. The images were acquired with a NIKON DXN1200F digital camera, which was controlled by the ECLIPSENET SOFTWARE (version 1.20.0 build 61).

### Cell transfection and RNA interference

Cells were transfected with the corresponding small interfering RNA (siRNA) using Lipofectamine RNAiMAX lipid reagent (INVITROGEN) as per manufacturer's instructions and as previously described^18,53^. Briefly, 2 × 10^5^ cells were plated unicellular on Vitronectin-coated 24-well dishes, grown 24 h with E8 media and then transfected with Silencer Select Negative Control #2 (AMBION, Cat. #4390846) or Silencer Select Validated HIF-1α siRNA (AMBION, Cat. #4390824, siRNA ID: s6539) or Silencer Select Validated EPAS1 (HIF-2α) siRNA (AMBION, Cat. #4390824, siRNA ID: s4699) or Silencer Select Validated BNIP-3 siRNA (AMBION, Cat. #4392420, siRNA ID: s2059) or Silencer Select PMAIP1 (NOXA) siRNA (AMBION, Cat. #4392420, siRNA ID: s10709) or Silencer Validated P53 siRNA (AMBION, Cat. # AM51331, siRNA ID: 106141). The concentration of siRNA used for cell transfection was 20 nM.

### Statistical analysis

Data were analyzed using GRAPHPAD PRISM VERSION 6. All results are expressed as mean ± SEM. One-way ANOVAs followed by Dunnett's multiple comparisons tests or two-tailed Student´s t-test were used to detect significant differences (*p* < 0.05) among treatments as indicated^18^.

## Supplementary information

Supplementary Information.

## Data Availability

The datasets generated during and/or analyzed during the current study are available from the corresponding author on reasonable request.
