# Supplementary material for: Chemical hypoxia induces apoptosis of human pluripotent stem cells by a NOXA-mediated HIF-1α and HIF-2α independent mechanism
Source: Sci Rep. 2020 Nov 26;10:20653. doi: 10.1038/s41598-020-77792-7 (PMC7692563; doi:10.1038/s41598-020-77792-7)
Supplement: Supplementary file 1 — Supplementary Information. [file 41598_2020_77792_MOESM1_ESM.pdf]

Supplementary information

Full Title: *Chemical hypoxia induces apoptosis of human pluripotent stem cells by a NOXA-mediated HIF-1α and HIF-2α independent mechanism*

Authors: Luciana Isaja[1], Sofía Mucci[1], Jonathan Vera, María Soledad Rodríguez-Varela, Mariela Marazita, Olivia Morris-Hanon, Guillermo Agustín Videla-Richardson, Gustavo Emilio Sevlever, María Elida Scassa and Leonardo Romorini.

[1] Both authors contributed equally to this work.

Author affiliations: Laboratorios de Investigación Aplicada en Neurociencias (LIAN-CONICET), Fundación para la Lucha contra las Enfermedades Neurológicas de la Infancia (Fleni), Ruta 9, Km 52.5, Belén de Escobar, Provincia de Buenos Aires, B1625XAF, Argentina.

Supplementary methods:

Antibodies and primers used:

	Primer sequence (5' → 3')	
**Name**	**Forward**	**Reverse**
RPL7	AATGGCGAGGATGGCAAG	TGACGAAGGCGAAGAAGC
BNIP-3	ATCGCCGCAGTTGCCCTCTGG	ATAGAAACCGAGGCTGGAACGCTG
BNIP-3L	AATGTCGTCCCACCTAGTCG	CCCCCATTTTTCCCATTGCC
MCL-1	GGGCAGGATTGTGACTCTCATT	GATGCAGCTTTCTTGGTTTATGG
PUMA	GACCTCAACGCACAGTACGAG	AGGAGTCCCATGATGAGATTGT
NOXA	ACCAAGCCGGATTTGCGATT	ACTTGCACTTGTTCCTCGTGG
P53	CAATAGGTGTGCGTCAGAAG	CTTACATCTCCCAAACATCCC
HIF-1α	ACCCACCGCTGAAACGCCAA	GGTGTCTGATCCTGAATCTGGGGC
EPAS1 (HIF-2α)	AAAACGAGTCCGAAGCCGAA	TGACAGAAAGATCATGTCGCCA

Supplementary Table S1. *Primers used for RT-qPCR experiments.*

**Antibody**	**Specie**	**Brand**	**N° Catalogue**	**Dilution**
α-HIF-1α	Monoclonal-Mouse	BD	610958	1/1000
α-HIF-2α	Polyclonal-Rabbit	Abcam	ab199	1/1000
α-CASPASE-9	Polyclonal-Rabbit	Cell Signaling	9502	1/1000
α-active CASPASE-3	Polyclonal-Rabbit	Abcam	ab13847	1/1000
α-PARP	Monoclonal-Mouse	Santa Cruz	sc-8007	1/1000
α-MCL-1	Monoclonal-Rabbit	Cell Signaling	94296	1/1000
α-BNIP-3	Monoclonal-Mouse	Santa Cruz	sc-56167	1/1000

α-BCL-X_L	Polyclonal-Rabbit	Santa Cruz	sc-634	1/1000
α-PUMA	Monoclonal-Rabbit	Abcam	ab33906	1/1000
α-NOXA	Monoclonal-Rabbit	Cell Signaling	14766	1/1000
α-P53	Monoclonal-Mouse	Abcam	ab1101	1/1000
α-ACTIN	Polyclonal-Goat	Santa Cruz	sc-1616	1/1000
α-GAPDH	Monoclonal-Mouse	Santa Cruz	sc-365062	1/2000

Supplementary Table S2. *Primary antibodies used for Western Blot and immunofluorescence experiments.*

	A	B	C	D	E	F	G	H	I	J	K	L	M	N
1	+	+	-	-	-	Blank	Blank	BAD	BAX	BCL-2	BCL-w	BID	CASP-3	CASP-8
2	+	+	-	-	-	Blank	Blank	BAD	BAX	BCL-2	BCL-w	BID	CASP-3	CASP-8
3	CD40	CD40	cIAP-2	CYTO-C	DR6	FAS	FASL	Blank	HSP27	HSP60	HSP70	HTRA	IGF-I	IGF-II
4	CD40	CD40	cIAP-2	CYTO-C	DR6	FAS	FASL	Blank	HSP27	HSP60	HSP70	HTRA	IGF-I	IGF-II
5	IGFBP-1	IGFBP-2	IGFBP-3	IGFBP-4	IGFBP-5	IGFBP6	IGF-1sR	LIVIN	P21	P27	P53	SMAC	SURVIVIN	sTNF-R1
6	IGFBP-1	IGFBP-2	IGFBP-3	IGFBP-4	IGFBP-5	IGFBP-6	IGF-1sR	LIVIN	P21	P27	P53	SMAC	SURVIVIN	sTNF-R1
7	sTNF-R2	TNF-α	TNF-α	TRAILR1	TRAILR2	TRAILR3	TRAILR4	XIAP	Blank	Blank	-	-	-	+
8	sTNF-R2	TNF-α	TNF-α	TRAILR1	TRAILR2	TRAILR3	TRAILR4	XIAP	Blank	Blank	-	-	-	+

Supplementary Table S3. *Apoptosis protein array map. Exact position in the membrane of 43 human related-apoptosis proteins, blanks and positive/negative controls is shown.*

Supplementary figures:

Chemical hypoxia induction by $CoCl_2$ and DMOG

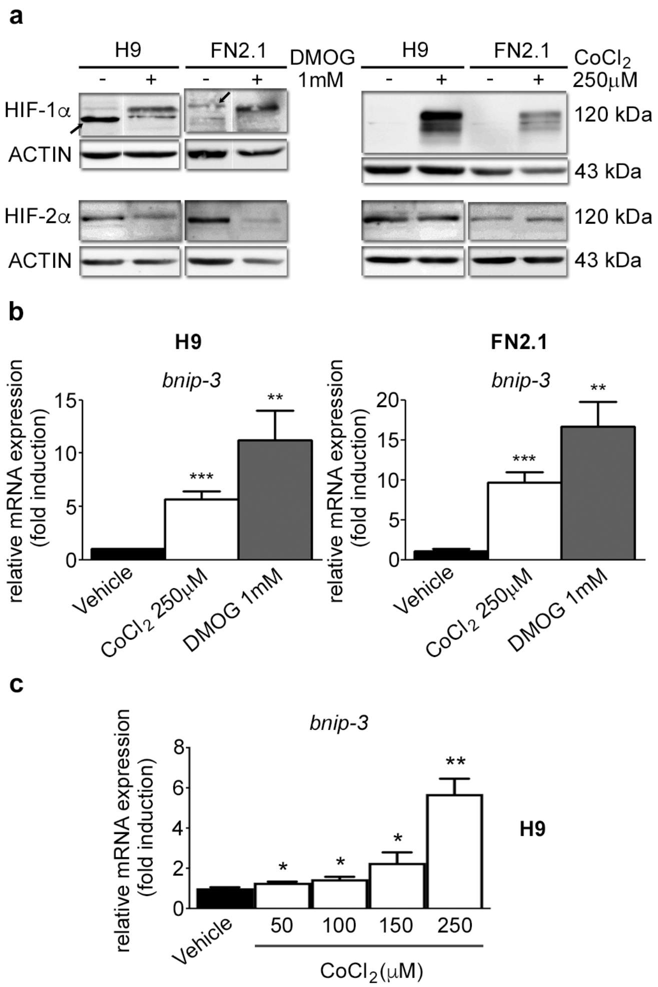

Supplementary Figure S1. ***Chemical hypoxia induction by $CoCl_2$ and DMOG.*** **(a)** *HIF-1α* and *EPAS1* (*HIF-2α*) protein expression levels were quantified by Western blot in H9 hESCs and FN2.1 hiPSCs upon DMOG (1mM for 24 hours) and $CoCl_2$ (250μM for 24 hours) treatments. ACTIN was used as loading control. Representative blots of three independent experiments are shown (full-length images are presented in Supplementary Fig. S14. In the case of HIF-1a and DMOG treatment, lanes were grouped and a white line was left dividing them). Black arrows: nonspecific bands. **(b)** Analysis of mRNA expression levels of *BNIP-3* quantified by RT-qPCR in FN2.1 and H9 hPSCs at 24 hours post $CoCl_2$ (250μM) and DMOG (1mM) treatments. *RPL7* mRNA expression levels were used as normalizer. Graphs show mean + SEM mRNA fold induction relative to Vehicle (DMSO treated) control cells

(arbitrarily set as 1) of at least three independent experiments. Statistical analysis was done by Student's t-test, (**) $p<0.01$ and (***) $p<0.001$ vs. Vehicle. **(c)** Analysis of mRNA expression levels of *BNIP-3* quantified by RT-qPCR in H9 hPSCs at 24 hours post $CoCl_2$ (50, 100, 150 and 250µM) treatment. *RPL7* mRNA expression levels were used as normalizer. Graphs show mean + SEM mRNA fold induction relative to Vehicle (H_2O treated) control cells (arbitrarily set as 1) of three independent experiments. Statistical analysis was done by Student's t-test, (*) $p<0.05$ and (**) $p<0.01$ vs. Vehicle.

CASPASE-3 activation by immunofluorescence upon chemical hypoxia induction

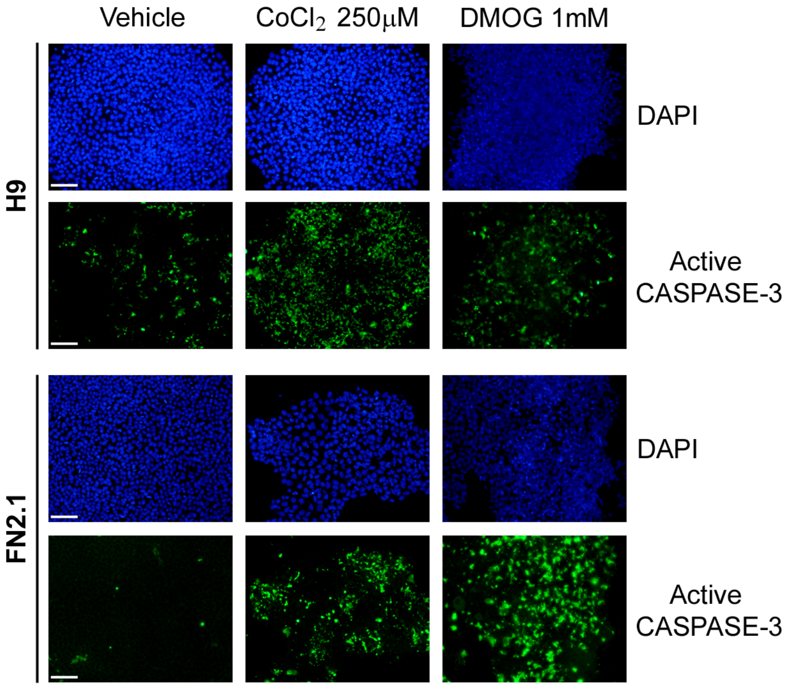

Supplementary Figure S2. ***CASPASE-3 activation upon chemical hypoxia induction.*** Representative micrographs of H9 and FN2.1 hPSCs immunostained against active CASPASE-3 (green) after 6 hours treatment with $CoCl_2$ (250µM) and DMOG (1mM). Figure shows representative images. The nuclei were counterstained with DAPI. Vehicle: DMSO. Scale bars represent 100 µm.

Quantification of Figure 3a western blot images

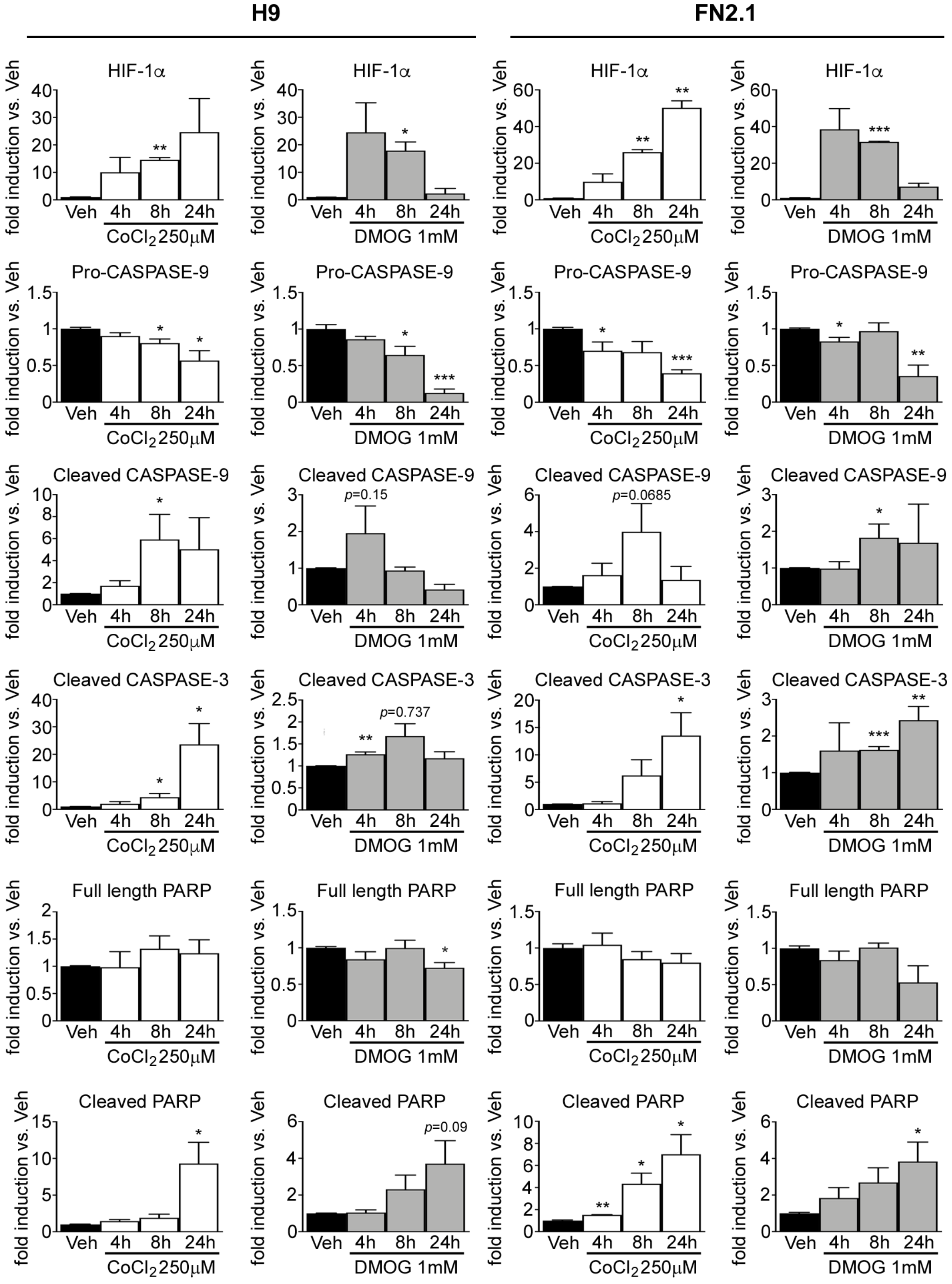

Supplementary Figure S3. ***Quantification of Figure 3a western blot images.*** Bar graphs represent densitometric quantification of bands of western blot images shown in Figure 3a. Veh: Vehicle. Data are expressed as means +

SEM fold induction relative to Vehicle (H_2O for $CoCl_2$ treatments and DMSO for DMOG treatments) (arbitrarily set as 1) and Statistical analysis was done by Student's t-test, (*) $p<0.05$, (**) $p<0.01$ and (***) $p<0.001$ vs. Vehicle.

Apoptotic protein profiling in chemical hypoxia-treated H9 hESCs

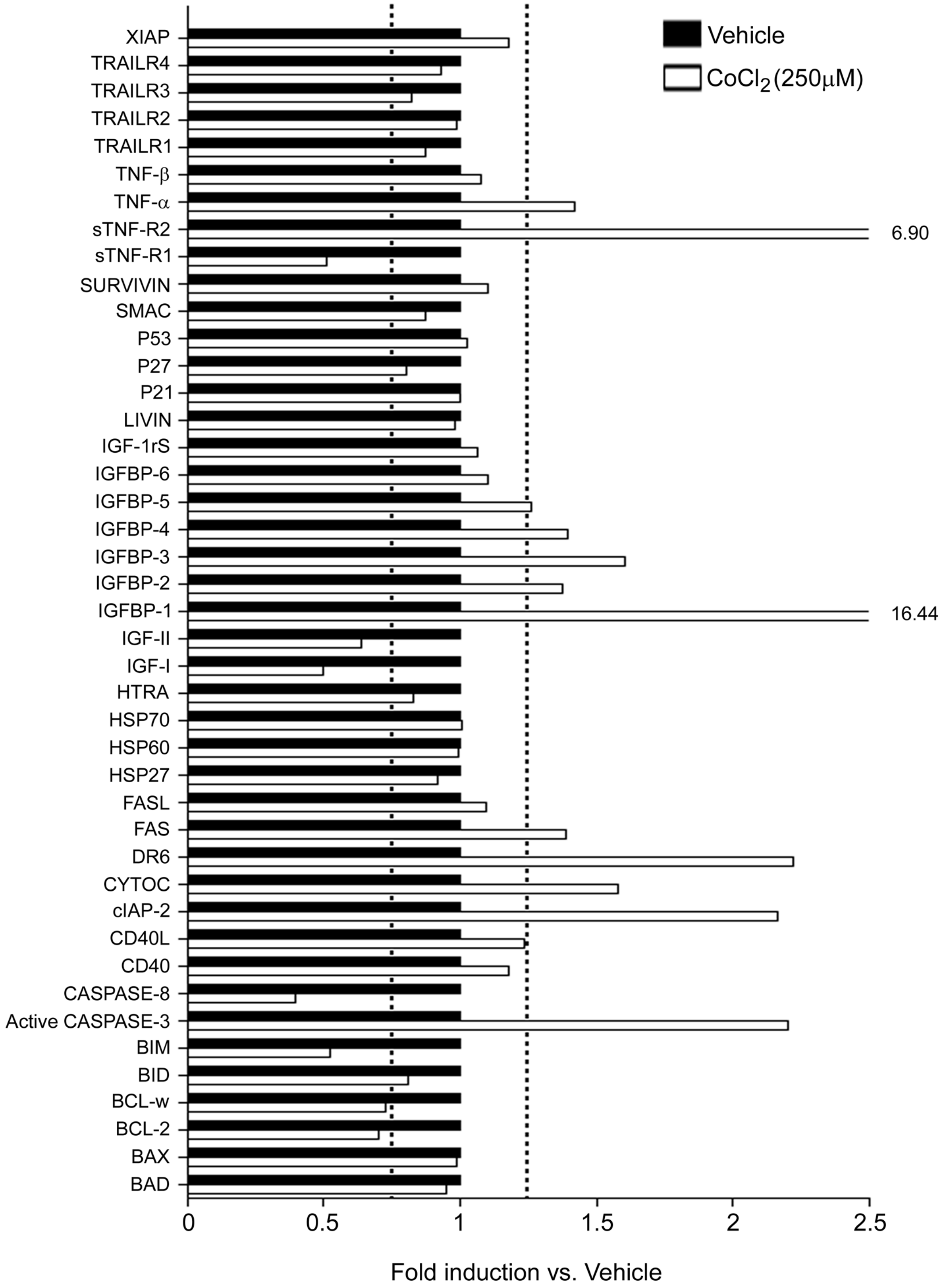

Supplementary Figure S4. ***Apoptotic protein profiling in $CoCl_2$-treated H9 hESCs.*** H9 hESCs were treated with $CoCl_2$ (250µM) for 8 hours. Graph shows pixel densities of 43 apoptosis-related proteins identified using a human Apoptosis Array Kit normalized against positive control dots and relativized against H_2O-treated (Vehicle) cells (arbitrary set a 1). 0.25-fold induction changes were arbitrary considered as differentially expressed proteins (Dotted line delimitation).

BNIP-3L, NOXA, MCL-1 and PUMA mRNA expression levels upon $CoCl_2$ treatment.

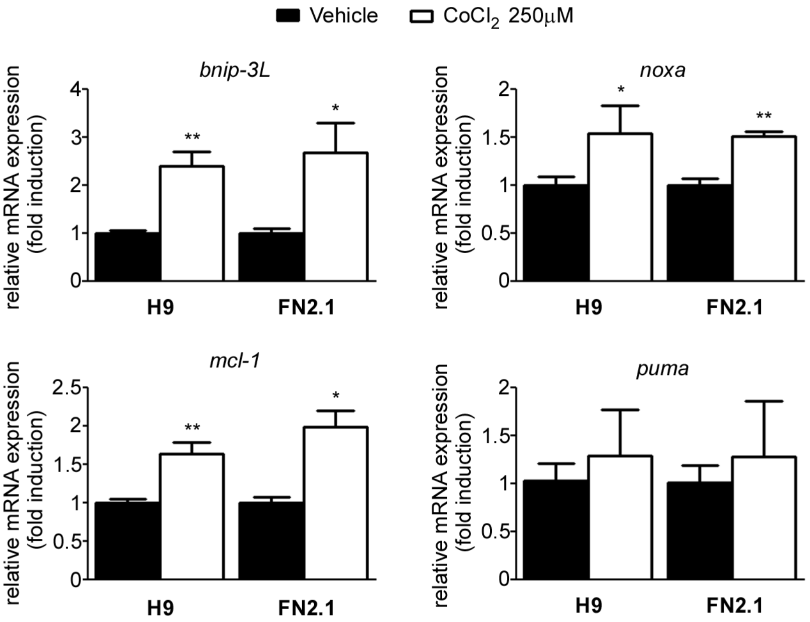

Supplementary Figure S5. ***BNIP-3L, NOXA, MCL-1 and PUMA mRNA expression levels upon $CoCl_2$ treatment.*** mRNA expression levels were analyzed by RT-qPCR in H9 and FN2.1 hPSCs at 24 hours post $CoCl_2$ (250µM) treatment with primers that amplified *bnip-3L, noxa, mcl-1* and *puma*. *RPL7* mRNA expression levels were used as normalizer. Graphs show mean + SEM mRNA fold induction relative to Vehicle (H_2O treated) control cells (arbitrarily set as 1) of at least three independent experiments. Statistical analysis was done by Student's t-test, (*) $p<0.05$ and (**) $p<0.01$ vs. Vehicle.

Quantification of PI staining cells

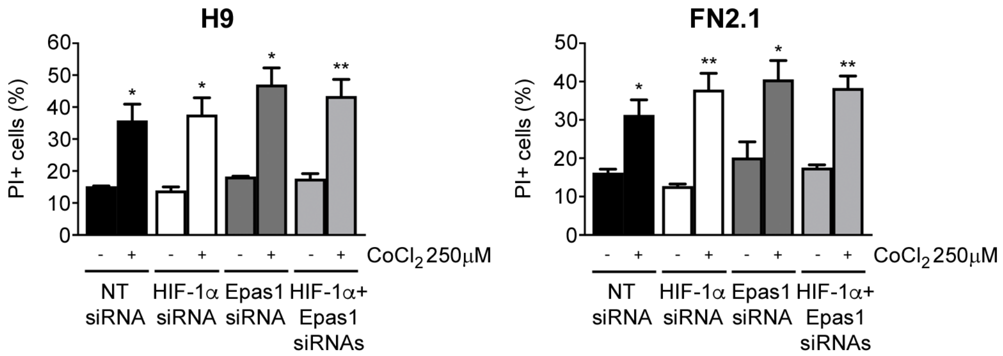

Supplementary Figure S6. ***HIF-1a and EPAS1 siRNAs: Graph and statistical analysis of percentage of PI positive cells upon $CoCl_2$ treatment.*** H9 hESCs and FN2.1 hiPSCs were transfected with negative control non-targeting siRNA (NT siRNA) (20nM), HIF-1α siRNA (20nM) and EPAS1 (HIF-2α) siRNA (20nM) and then mean + SEM of percentage of PI positive cells, determined by flow cytometric analysis at 24 hours post $CoCl_2$ (250μM), from three independent experiments is graphed. Statistical analysis was done by Student's t-test, (*) $p<0.05$ and (**) $p<0.01$ vs. Vehicle (H_2O).

Involvement of HIF-1α and HIF-2α in DMOG-induced apoptosis in hPSCs.

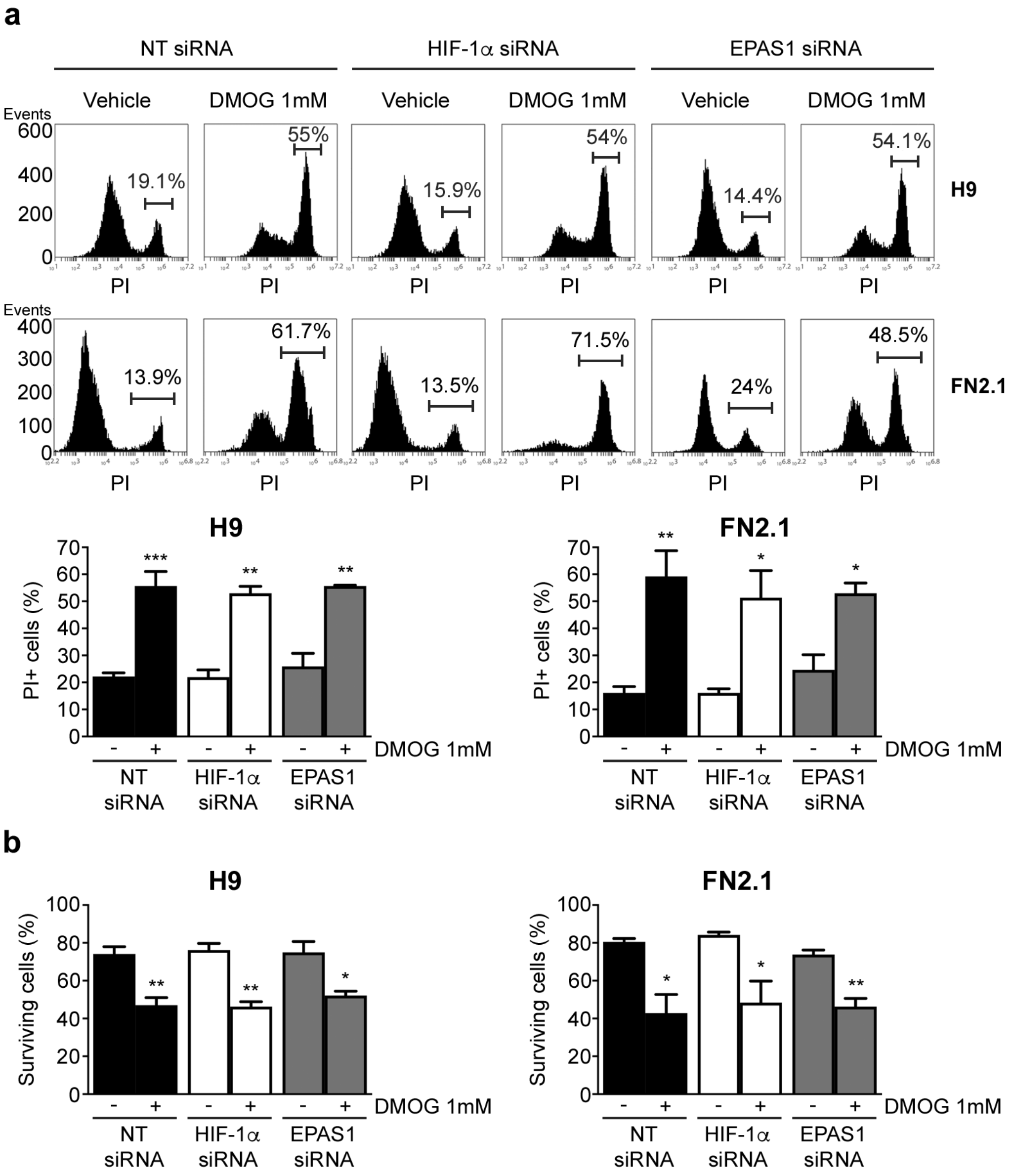

Supplementary Figure S7. ***Effect of siRNA-mediated down regulation of HIF-1α and HIF-2α in hPSCs cell viability and death upon DMOG treatment.*** H9 hESCs and FN2.1 hiPSCs were transfected with negative control non-targeting siRNA (NT siRNA) (20nM), HIF-1α siRNA (20nM) and EPAS1 (HIF-2α) siRNA (20nM) and then: **(a)** Representative histograms of Propidium iodide (PI) stained H9 and FN2.1 unfixed cells at 48 hours post siRNA transfection. Chemical hypoxia was induced with DMOG (1mM) at 24 hours post siRNA transfection. Percentage of PI positive cells (late apoptotic or necrotic) was determined by flow cytometric analysis. Vehicle: DMSO. Mean + SEM of percentage of PI positive cells from three independent experiments is graphed. Statistical analysis was done

by Student's t-test, (*) $p<0.05$, (**) $p<0.01$ and (***) $p<0.001$ vs. Vehicle (DMSO). **(b)** Histograms show percentage of surviving cells assessed by Trypan blue exclusion method at 48 hours post siRNA transfection. 24 hours after transfection cells were treated with DMOG (1mM). Mean + SEM from three independent experiments are shown. Statistical analysis was done by Student's t-test, (*) $p<0.05$ and (**) $p<0.01$ vs. NT siRNA.

Involvement of BNIP-3 in $CoCl_2$-induced apoptosis in hPSCs.

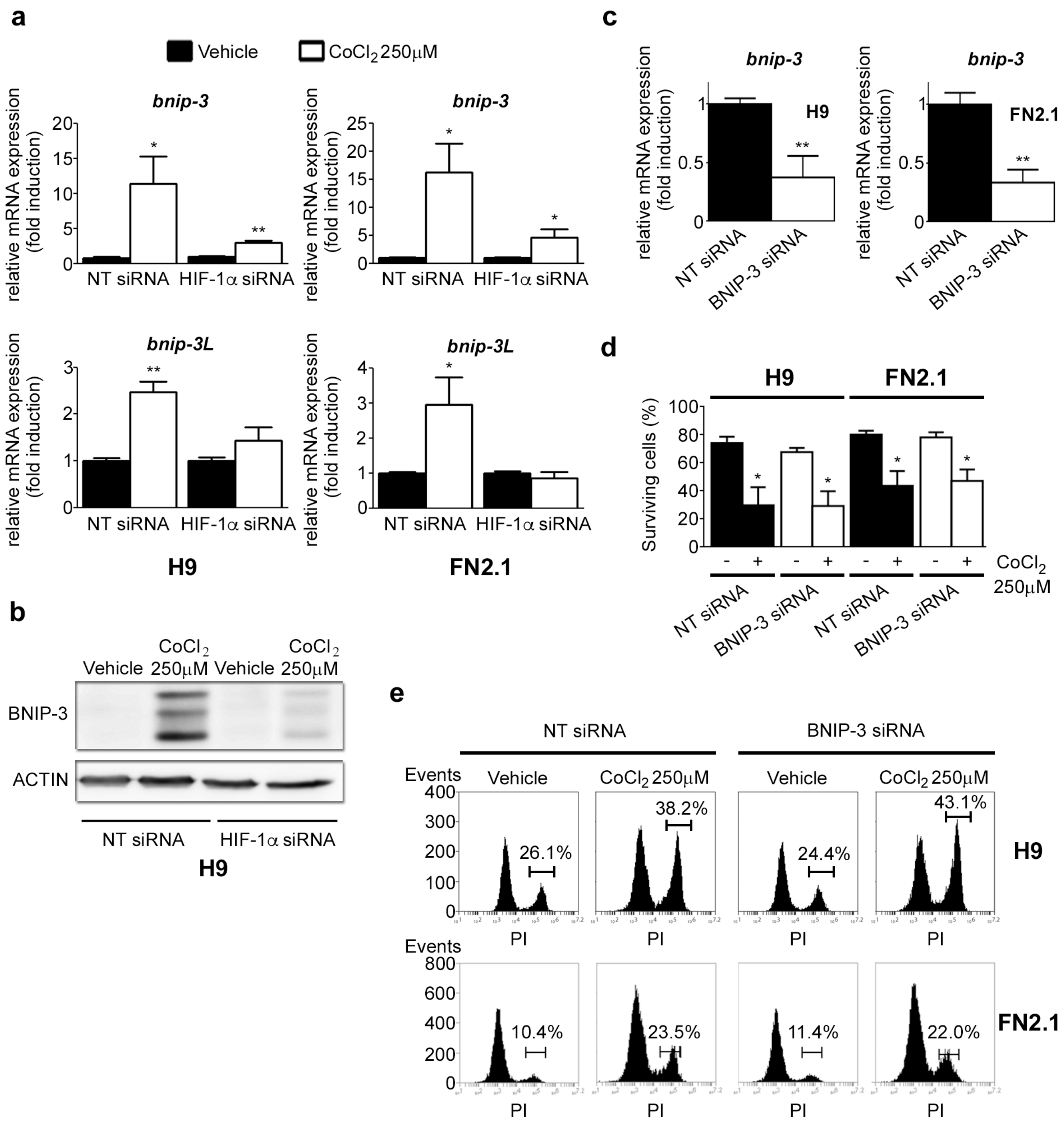

Supplementary Figure S8. ***Involvement of BNIP-3 in $CoCl_2$-induced apoptosis in hPSCs.*** H9 hESCs and FN2.1 hiPSCs were transfected with negative control non-targeting siRNA (NT siRNA) (20nM) or HIF-1α (20nM) or BNIP-3 (20nM) and then: **(a)** mRNA expression levels of *BNIP-3* and *BNIP-3L* were analyzed by RT-qPCR at 48 hours post siRNAs transfection. 24 hours after transfection cells were treated with $CoCl_2$ (250µM). *RPL7* mRNA expression levels were used as normalizer. Graph shows mean + SEM mRNA fold induction relative to Vehicle (H_2O) arbitrarily set as 1 from three independent experiments. Statistical analysis was done by Student's t-test, (*) $p<0.05$ and (**) $p<0.01$ vs. Vehicle. **(b)** Expression levels of BNIP-3 were analyzed by Western blot in H9 cells at 48 hours post siRNAs transfection. 24 hours after transfection cells were treated with $CoCl_2$ (250µM). ACTIN was used as loading control. Representative blots of three independent experiments are shown (full-length images are presented in Supplementary Fig. S15). **(c)** mRNA expression levels of *BNIP-3* were analyzed by RT-qPCR at 48 hours post siRNAs transfection. *RPL7* mRNA expression levels were used as normalizer. Graph shows mean + SEM mRNA fold induction relative to NT siRNA transfectants arbitrarily set as 1 from three independent experiments. Statistical analysis was done by Student's t-test, (**) $p<0.01$ vs. NT siRNA. **(d)** Histograms show percentage of surviving cells assessed by Trypan blue exclusion method at 48 hours post siRNA transfection. 24 hours after transfection cells were treated with $CoCl_2$ (250µM). Mean + SEM from at least three independent experiments are shown. Statistical analysis was done by Student's t-test, (*) $p<0.05$ vs. NT siRNA. **(e)** Representative histograms, of three independent experiments, of Propidium iodide (PI) stained H9 and FN2.1 unfixed cells at 48 hours post siRNA transfection. Chemical hypoxia was induced with $CoCl_2$ (250µM) at 24 hours post siRNA transfection. Percentage of PI positive cells (late apoptotic or necrotic) was determined by flow cytometric analysis. Vehicle: H_2O.

Effect of siRNA-mediated down regulation of P53 and NOXA in hPSCs cell viability and death upon DMOG treatment

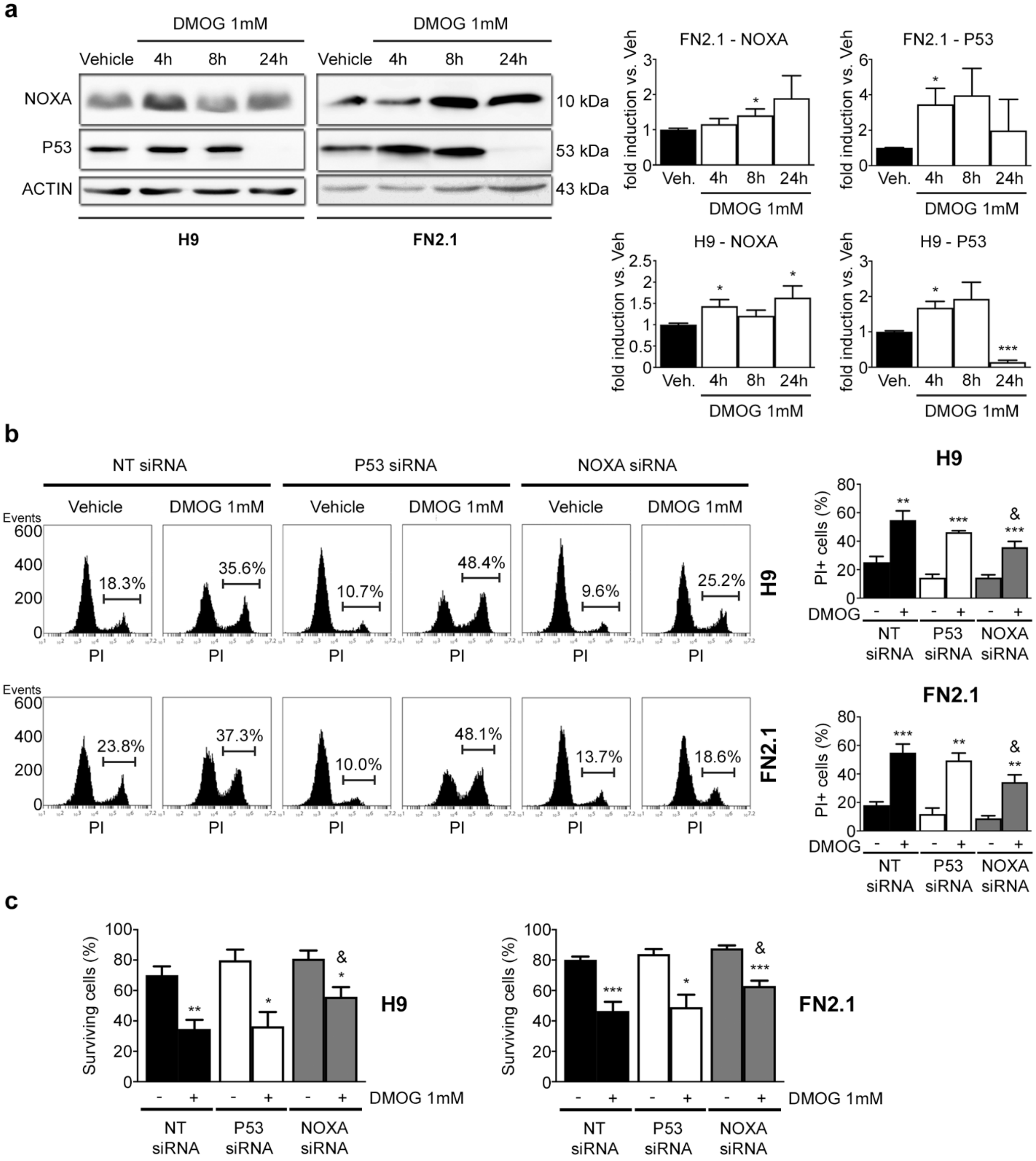

Supplementary Figure S9. ***Effect of siRNA-mediated down regulation of P53 and NOXA in hPSCs cell viability and death upon DMOG treatment*****. (a)** Expression levels of NOXA and P53 were analyzed by Western blot in H9

and FN2.1 cells at 4, 8 and 24 hours post DMOG (1mM) treatment. ACTIN was used as loading control. Representative blots of three independent experiments are shown (full-length images are presented in Supplementary Fig. S16). Bar graphs represent densitometric quantification of bands. Data are expressed as means + SEM fold induction relative to Vehicle (DMSO) (arbitrarily set as 1) and Statistical analysis was done by Student's t-test, (*) $p<0.05$ and (***) $p<0.001$ vs. Vehicle (DMSO). **(b; c)** H9 hESCs and FN2.1 hiPSCs were transfected with negative control non-targeting siRNA (NT siRNA) (20nM) or P53 siRNA (20nM) or NOXA siRNA (20nM) and then: **(b)** Representative histograms of Propidium iodide (PI) stained H9 and FN2.1 unfixed cells at 48 hours post siRNA transfection. Chemical hypoxia was induced with DMOG (1mM) at 24 hours post siRNA transfection. Percentage of PI positive cells (late apoptotic or necrotic) was determined by flow cytometric analysis. Vehicle: DMSO. Mean + SEM from at least three independent experiments are shown. Statistical analysis was done by Student's t-test, (**) $p<0.01$ and (***) $p<0.001$ vs. NT siRNA; (&) $p<0.05$ vs. NT + DMOG 1mM. **(c)** Bar graphs show percentage of surviving cells assessed by Trypan blue exclusion method at 48 hours post siRNA transfection. 24 hours after transfection cells were treated with DMOG (1mM). Mean + SEM from at least three independent experiments are shown. Statistical analysis was done by Student's t-test, (*) $p<0.05$, (**) $p<0.01$ and (***) $p<0.001$ vs. NT siRNA; (&) $p<0.05$ vs. NT + DMOG 1mM.

Full Western blot images of Figure 3

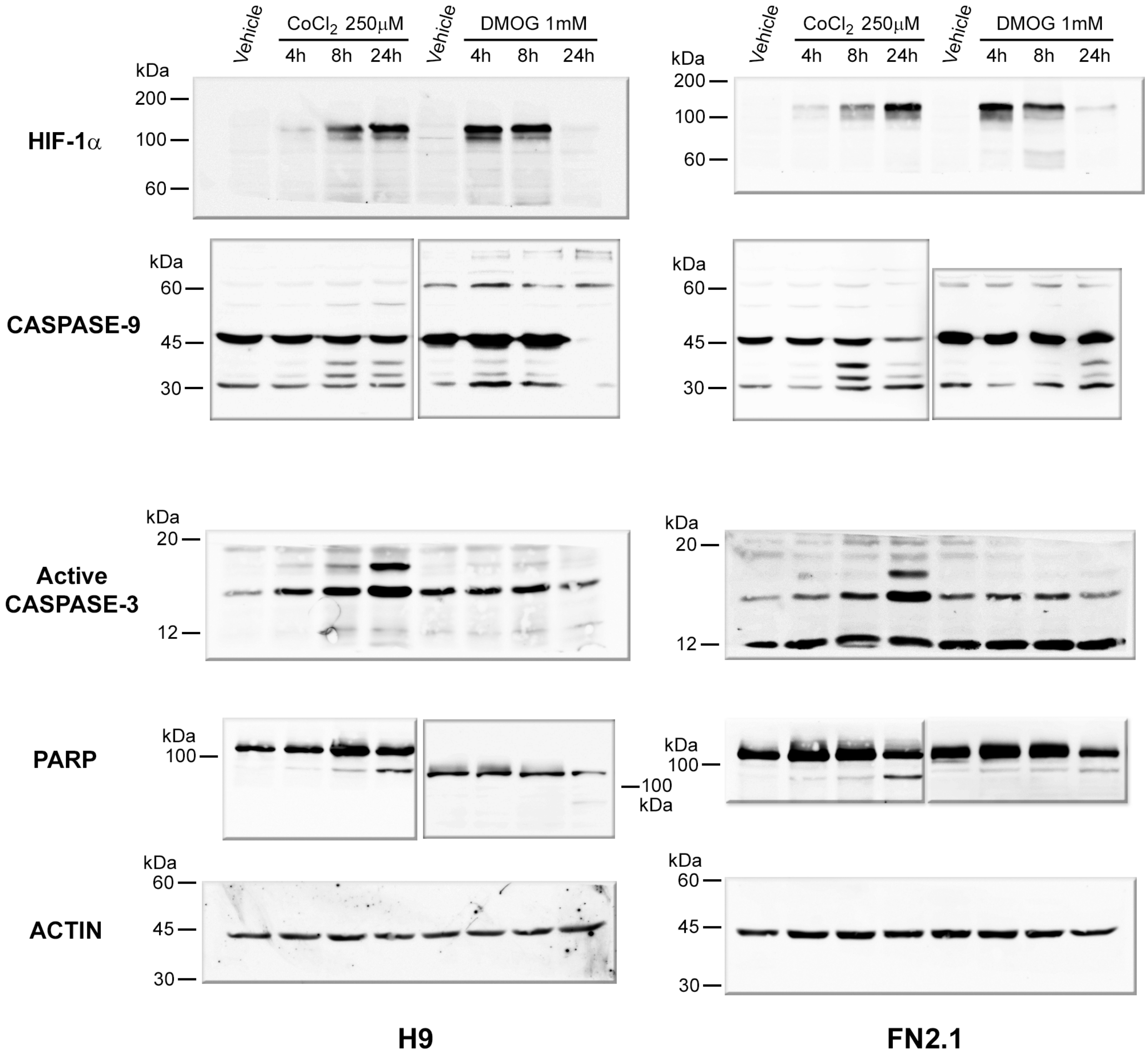

Supplementary Figure S10. ***Full Western blot images of Figure 3: CASPASE-9, CASPASE-3 activation and PARP cleavage and apoptotic protein profiling upon chemical hypoxia induction.*** Cleavage and activation of initiator CASPASE-9, effector CASPASE-3, PARP proteolysis (CASPASE-3 substrate) and HIF-1α stabilization were analyzed by Western blot in H9 hESCs and FN2.1 hiPSCs at 4-, 8- and 24-hours post $CoCl_2$ (250μM) and DMOG (1mM) treatments. ACTIN was used as loading control. In all cases ColorBurst Electrophoresis Marker from Sigma (C1992) was used.

Full Western blot images of Figure 4

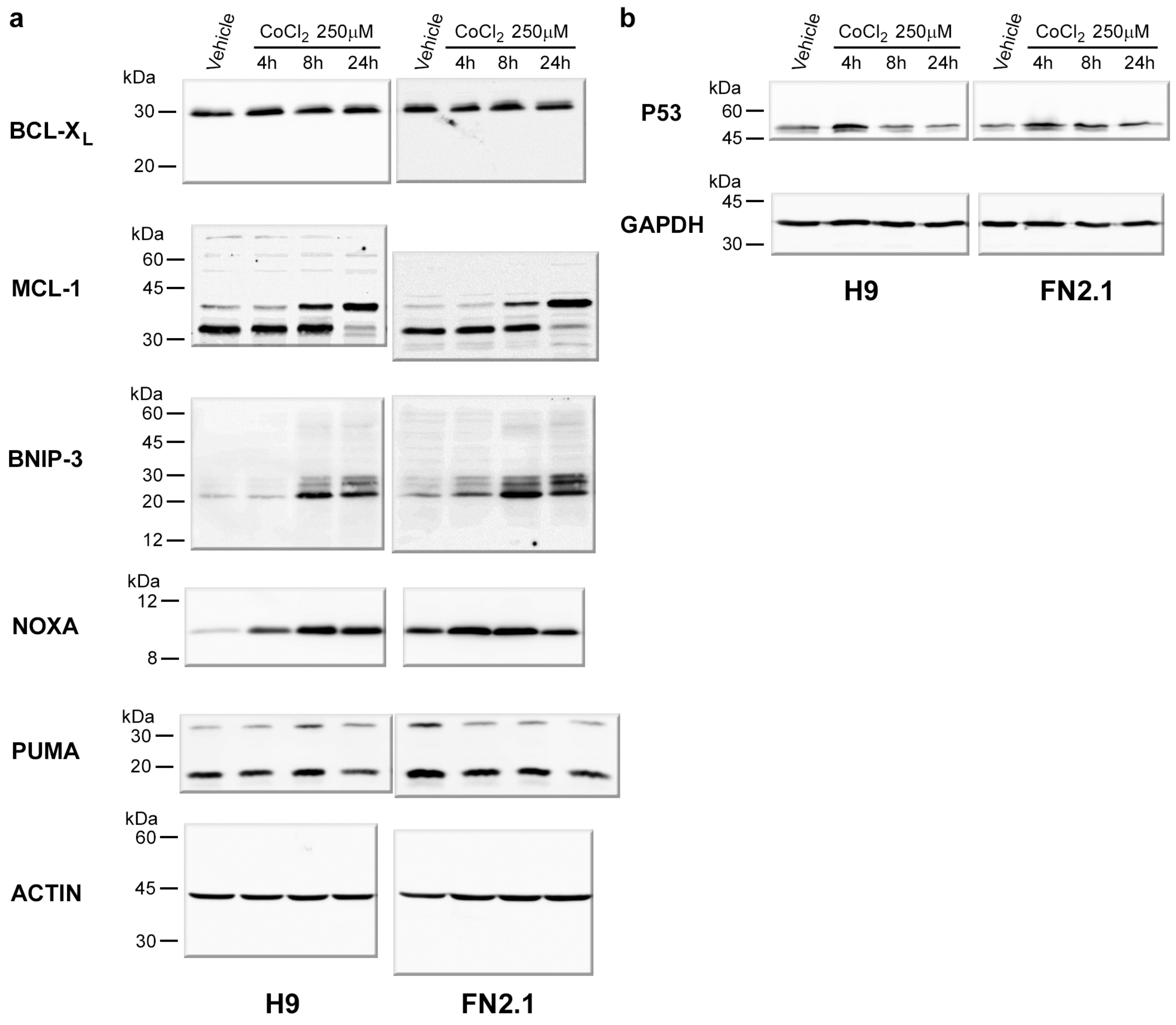

Supplementary Figure S11. ***Full Western blot images of Figure 4: BCL-2 family members and P53 expression levels.*** Expression levels of **(a)** BCL-2 family members, including BCL-X_L (anti-apoptotic), MCL-1 (anti-apoptotic), BNIP-3 (pro-apoptotic), NOXA (pro-apoptotic) and PUMA (pro-apoptotic) or **(b)** P53 were analyzed by Western blot in H9 and FN2.1 cells at 4, 8 and 24 hours post $CoCl_2$ (250μM) treatment. ACTIN or GAPDH were used as loading control. In all cases ColorBurst Electrophoresis Marker from Sigma (C1992) was used.

Full Western blot images of Figure 5

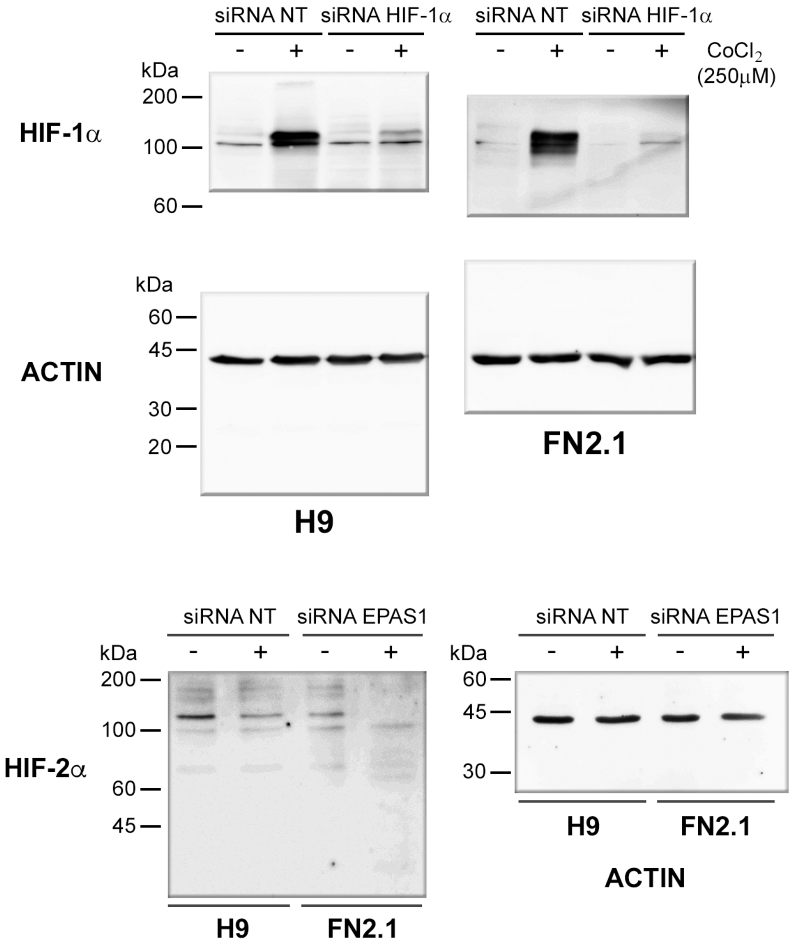

Supplementary Figure S12. ***Full Western blot images of Figure 5: Effect of siRNA-mediated down regulation of HIF-1α and EPAS1 (HIF-2α) in hPSCs cell viability and death upon chemical hypoxia induction.*** H9 hESCs and FN2.1 hiPSCs were transfected with negative control non-targeting siRNA (NT siRNA) (20nM) or HIF-1α siRNA (20nM) or EPAS1 siRNA (20nM) and then expression levels of HIF-1α or HIF-2α (EPAS1) were analyzed by Western blot in H9 and FN2.1 cells at 48 hours post siRNAs transfection. HIF-1α was stabilized with $CoCl_2$ (250μM for 24 hours) treatment. ACTIN was used as loading control. In all cases ColorBurst Electrophoresis Marker from Sigma (C1992) was used.

Full Western blot images of Figure 6

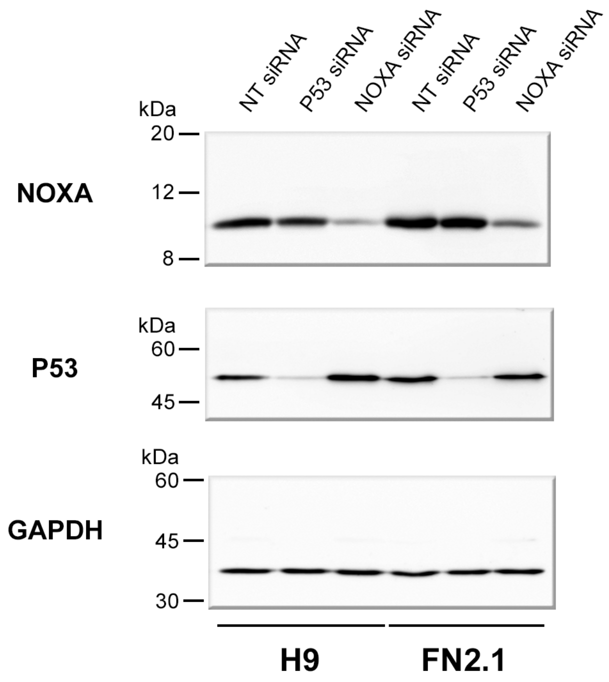

Supplementary Figure S13. ***Full Western blot images of Figure 6: Effect of siRNA-mediated down regulation of P53 and NOXA in hPSCs cell viability and death upon chemical hypoxia induction.*** H9 hESCs and FN2.1 hiPSCs were transfected with negative control non-targeting siRNA (NT siRNA) (20nM) or P53 siRNA (20nM) or NOXA siRNA (20nM) and then expression levels of NOXA and P53 were analyzed by Western blot in H9 and FN2.1 cells at 48 hours post siRNAs transfection. GAPDH was used as loading control. In all cases ColorBurst Electrophoresis Marker from Sigma (C1992) was used.

Full Western blot images of Supplementary Figure S1

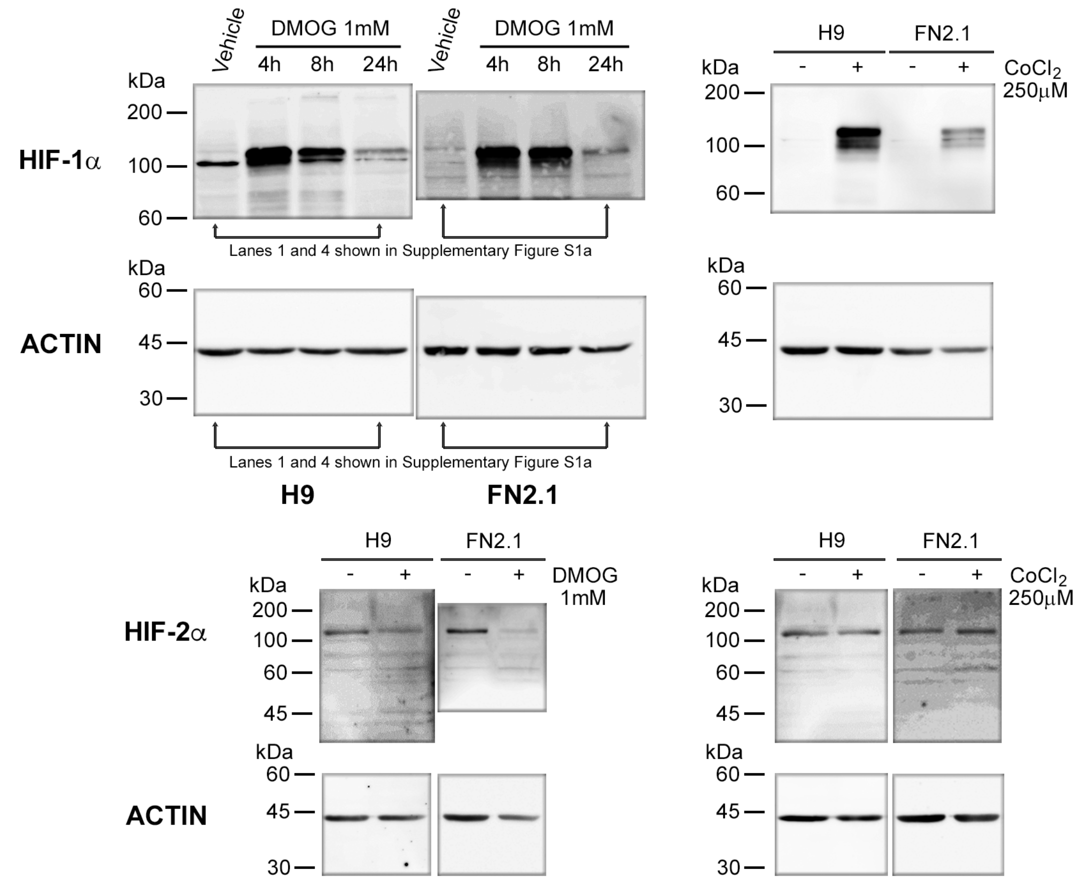

Supplementary Figure S14. ***Full western blot images of Figure Supplementary S1: Chemical hypoxia induction by $CoCl_2$ and DMOG.*** **(a)** *HIF-1α* and *EPAS1* (*HIF-2α*) protein expression levels were quantified by Western blot in H9 hESCs and FN2.1 hiPSCs upon DMOG (1mM for 24 hours) and $CoCl_2$ (250μM for 24 hours) treatments. ACTIN was used as loading control. In all cases ColorBurst Electrophoresis Marker from Sigma (C1992) was used. Arrows indicate the lanes (1 and 4) that are shown in Supplementary Figure S1 in the case of HIF-1α/ACTIN for DMOG treatment. The rest of the lanes in these gels, although they show an increase in the expression levels of HIF-1α, were cut from the gel to unify criteria and only show the expression levels at 24 hours of treatment. Lanes 1 and 4 were grouped and a white line was left dividing them.

Full Western blot images of Supplementary Figure S8

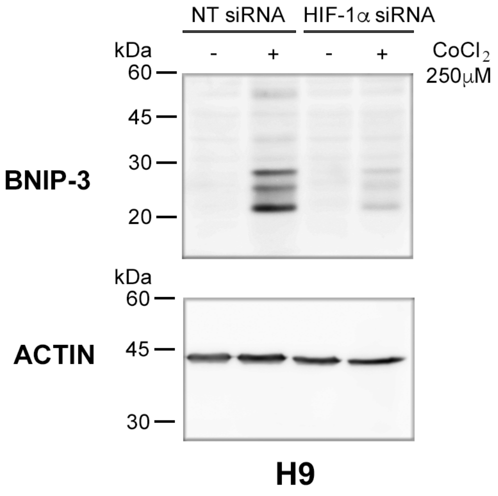

Supplementary Figure S15. ***Full western blot images of Figure Supplementary S8: Involvement of BNIP-3 in $CoCl_2$-induced apoptosis in hPSCs.*** H9 hESCs and FN2.1 hiPSCs were transfected with negative control non-targeting siRNA (NT siRNA) (20nM) or HIF-1α (20nM) or BNIP-3 (20nM) and then expression levels of BNIP-3 were analyzed by Western blot in H9 cells at 48 hours post siRNAs transfection. 24 hours after transfection cells were treated with $CoCl_2$ (250μM). ACTIN was used as loading control. In all cases ColorBurst Electrophoresis Marker from Sigma (C1992) was used.

Full Western blot images of Supplementary Figure S9

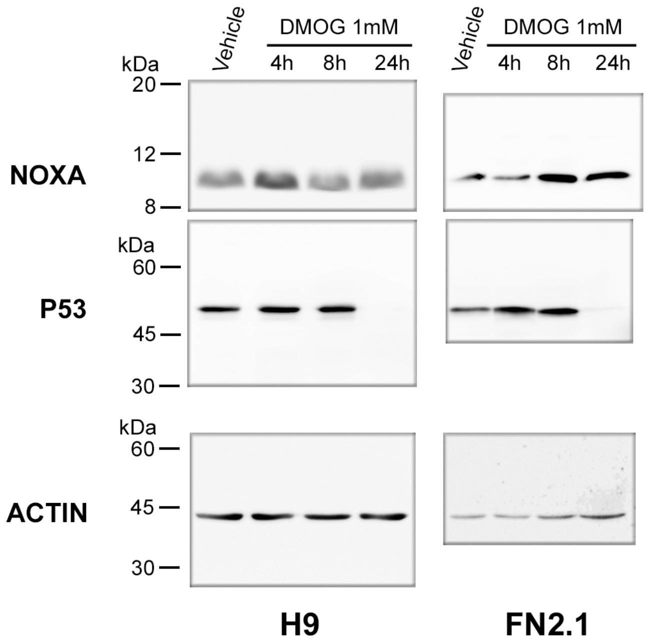

Supplementary Figure S16. ***Full western blot images of Figure Supplementary S9: Effect of siRNA-mediated down regulation of P53 and NOXA in hPSCs cell viability and death upon DMOG treatment***. Expression levels of NOXA and P53 were analyzed by Western blot in H9 and FN2.1 cells at 4, 8 and 24 hours post DMOG (1mM) treatment. ACTIN was used as loading control. In all cases ColorBurst Electrophoresis Marker from Sigma (C1992) was used.
